# Inertial Sensors to Assess Gait Quality in Patients with Neurological Disorders: A Systematic Review of Technical and Analytical Challenges

**DOI:** 10.3389/fpsyg.2017.00817

**Published:** 2017-05-18

**Authors:** Aliénor Vienne, Rémi P. Barrois, Stéphane Buffat, Damien Ricard, Pierre-Paul Vidal

**Affiliations:** ^1^CNRS UMR 8257, Cognition and Action Group, Cognac-G, Université Paris Descartes, Service de Santé des ArméesParis, France; ^2^Institut de Recherche Biomédicale des Armées, Service de Santé des ArméesBrétigny-sur-Orge, France; ^3^Ecole du Val-de-Grâce, Service de Santé des ArméesParis, France; ^4^Service de Neurologie de l’Hôpital d’Instruction des Armées de Percy, Service de Santé des ArméesClamart, France

**Keywords:** gait analysis, gait disorders, wearable inertial sensors, inertial measurement unit, accelerometer

## Abstract

Gait disorders are major causes of falls in patients with neurological diseases. Understanding these disorders allows prevention and better insights into underlying diseases. InertiaLocoGraphy (ILG) –the quantification of gait by using inertial measurement units (IMUs) –shows great potential to address this public health challenge, but protocols vary widely and normative values of gait parameters are still unavailable. This systematic review critically compares ILG protocols, questions features extracted from inertial signals and proposes a semeiological analysis of clinimetric characteristics for use in neurological clinical routine. For this systematic review, PubMed, Cochrane and EMBASE were searched for articles assessing gait quality by using IMUs that were published from January 1, 2014 to August 31, 2016. ILG was used to assess gait in a wide range of neurological disorders – including Parkinson disease, mild cognitive impairment, Alzheimer disease, cerebral palsy, and cerebellar atrophy – as well as in the faller or frail older population and in people presenting rheumatological pathologies. However, results have not yet been driving changes in clinical practice. One reason could be that studies mainly aimed at comparing pathological gait to healthy gait, but there is stronger need for semiological descriptions of gait perturbation, severity or prognostic assessment. Furthermore, protocols used to assess gait using IMUs are too many. Likely, outcomes are highly heterogeneous and difficult to compare across large panels of studies. Therefore, homogenization is needed to foster the use of ILG to assess gait quality in neurological routine practice. The pros and cons of each protocol are emphasized so that a compromise can be reached. As well, analysis of seven complementary clinical criteria (springiness, sturdiness, smoothness, steadiness, stability, symmetry, synchronization) is advocated.

## Introduction

Walking is a complex activity and a permanent decision-making process that can be altered in a variety of neurological pathologies. Gait disorders can lead to impaired mobility, disability, fear of falling or falls, which can result in reduced quality of life and increased risk of death (Wilson et al., 2002; Jørstad et al., 2005; Snijders et al., 2007). Assessment of walking impairments could help predict falls (Barrois et al., 2017) which could be an argument for prevention and correction measures.

3D Inertial Measurement Units (IMUs), also called wearable inertial sensors, are widely used for assessing gait characteristics in both healthy people and those with abnormalities. Compared to other types of non-invasive sensors used to assess gait (video motion analysis or mat), 3D IMUs are small and light. Thus, gait evaluation with use of 3D IMUs, or InertiaLocoGraphy (ILG), suits both hour-long ambulatory measurements and evaluation in point-of-care environments (Maetzler et al., 2013; Del Din et al., 2016b). Nevertheless, recent studies have often focused on one pathology only, limiting the possibility to ponder the role of pathologies, when several could explain the clinical presentation. In addition, most studies have been restricted by their limited follow-up. Thus, endpoints are mainly descriptive and do not provide prognostic information. Therefore, a comprehensive overview of existing results is needed to delve further into the clinimetric characteristics of IMUs. Such a review was successfully performed for Parkinson’s disease (Maetzler et al., 2013, 2016; Hubble et al., 2015) and this article aims at including other neurological conditions that present altered gait.

Protocols designed to assess gait using IMUs are many, and the plethora of quantified outcomes is an obstacle for a comprehensive overview of pathological gaits, which can be highly confusing for the clinician (Graham et al., 2008; Lord et al., 2013). According to a recent panoramic review, only 6% of sensors (including IMUs and magnetometers) used to assess Parkinson disease are precise and efficient enough for clinical testing (Sánchez-Ferro et al., 2016).

This review intends to provide a more thorough assessment of the features of IMUs and present a more complete assessment of the set-up to add to already published reviews of gait in Parkinson disease.

## Materials and Methods

This review was registered with the International Prospective Register of Systematic Reviews on August 31, 2016 (Registration: CRD42016043555). Both the search strategy and study protocol are available at http://www.crd.york.ac.uk/PROSPEROFILES/43555_STRATEGY_20160802.pdf.

### Search Strategy

We searched MEDLINE via PubMed, Cochrane, and EMBASE electronic databases to identify articles published from January 1, 2014 to August 31, 2016 that described the analysis of gait quality by using inertial sensors in healthy older adults or people with any pathology. Searches were not limited to neurological patients so as to compare with other types of gait disorders. The search involved the key words “Gait” with “inertial measurement unit,” “IMU,” “inertial sensor,” “accelerometer” or “gyrometer.”

### Inclusion Criteria

We included articles of studies of humans that investigated gait with IMUs, computed parameters to quantify gait, included at least one group of ill or older people, and compared at least 2 cohorts of participants. We excluded articles of studies that quantified other activities (such as standing or running) or assessed general physical activity (step count or walking bout length); focused on walking segmentation or event detection (U-turn, freezing of gait); assessed gait from raw data or used IMUs as a feedback tool; or included only people younger than 65 years. The PRISMA guidelines were used to select articles.

### Review Process

Potentially eligible studies were screened for eligibility independently by 2 review authors (AV and DR) on the basis of the title and abstract for the human research criterion and main text for other criteria. Discrepancies between reviewers were discussed to reach consensus. Then, eligible articles underwent data extraction and quality assessment.

### Quality Assessment

We evaluated the quality of articles by using a 20-item quality checklist (details in Supplementary Material S1) for assessing gait studies that we adapted from Hubble’s 17-item quality checklist for longitudinal studies (Hubble et al., 2015). Each article was assessed by three reviewers (AV, DR, RM), each blinded to the score given by the two others. Disagreements were discussed to achieve agreement on final scores. Scores for each study were based on reporting, internal quality, external quality and power and were reported as percentages for each category, a low percentage indicating low quality and a high percentage, high quality (details in Supplementary Material S1).

With appraisal of methodology quality, 22 studies were identified as being at low methodological quality (from 33 to 48%), 49 at moderate methodological quality (from 50 to 69%), and 7 at high methodological quality (from 71 to 90%) (Supplementary Table S1). A similar distribution was found for neurology papers only. Generally, studies lacked arguments for power, which was rarely computed, and less than 80% power when computed *a posteriori*. Studies were also defective in representativeness of the sample (external validity) as well as controlling for confounding factors (internal validity).

## Results

**FIGURE 1:**
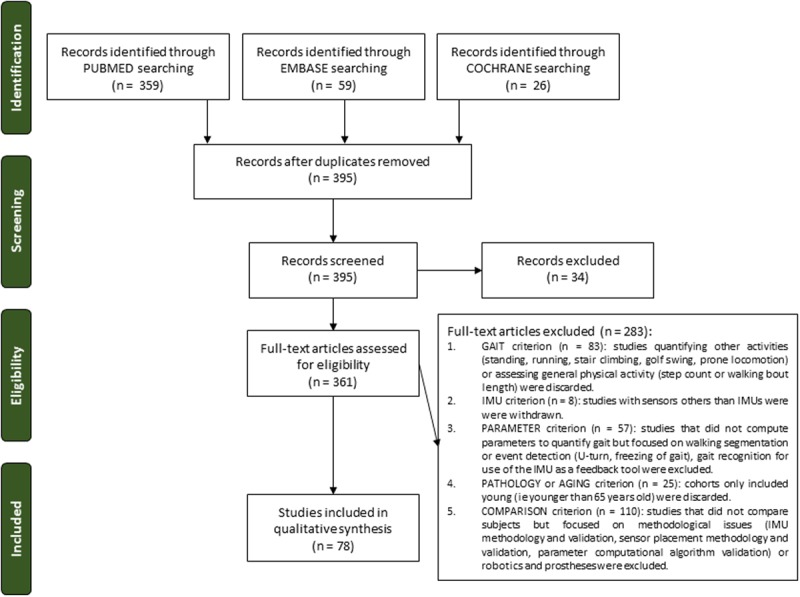
**PRISMA flow chart illustrating the study selection process resulting in 78 articles included in the review.** IMU, inertial measurement units.

In total, 78 full-text articles were selected (**Figure 1**). Detailed data as well as references to studies assessing gait in extraneurologic conditions can be found in Supplemental Material S3.

### Aims and Methods of ILG

#### Spectrum of Pathological Conditions Assessed

In total, 47.4% of all described assessment of neurological care. Other clinical domains were gerontological care (33.3%) and rheumatological care (19.2%) (Supplementary Material S2). One study assessed rapid weight loss after bariatric surgery.

**FIGURE 2:**
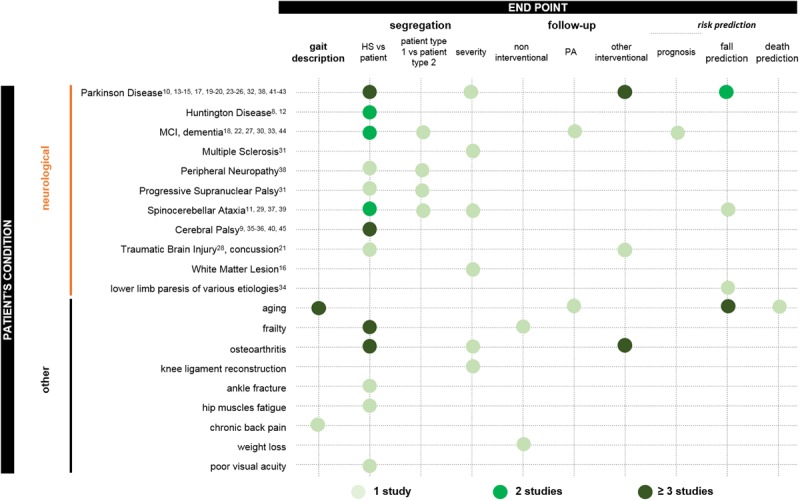
**Pathologies assessed in the included studies and gait analysis in patient care.** Dot colors specify the number of studies that addressed the issue. See figures for precise definition of colors.

For neurological patients as well as patients with other conditions, indications for gait quantification varied from early assessment (risk prediction for instance) to late severity assessment or treatment efficiency evaluation (**Figure 2**). Some studies included and compared several types of disorders: frailty and mild cognitive impairment; Parkinson disease and dementia (Yoneyama et al., 2016); Parkinson disease and peripheral neuropathy (Sejdic et al., 2014); or Parkinson disease and progressive supranuclear palsy (Hatanaka et al., 2016). Most of the other studies aimed at differentiating pathological and healthy participants. Protocols of gait evaluation that used 3D IMUs.

American and French neurology (Thurman et al., 2008; Gilman, 2010; Collége des Enseignants de Neurologie [CEN], 2016) as well as rheumatology and gerontology societies recommended the use of standardized measures to assess gait: the 6-min walk test (assesses exercise tolerance in frail older adults), the timed 25-foot walk test (a shorter test mainly used for patients with multiple sclerosis), and the Timed-Up-and-Go (TUG) test (Andrzejewski et al., 2016). Nonetheless, protocols offer endless variabilities at each phase of their design as described below.

##### Environment: laboratory versus ambulatory

**FIGURE 3:**
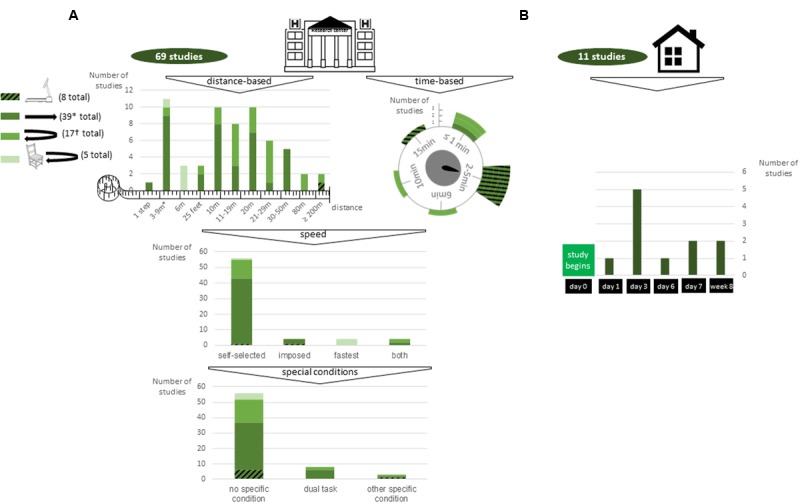
**Methods for gait assessment in (A)** laboratory and clinical settings and **(B)** ambulatory settings. Striped green: treadmill walking; dark green: unlevelled surface without U-turn; emerald green: unlevelled surface with U-turn; light green: stand up from a chair and unlevelled surface with U-turn. ^∗^2 studies were time-based but time limit was not specified; 1 study included 3 types of floor (flat floor, 8% and 20% slopes); 1 study included a trial with an obstacle. ^†^1 study analyzed only a large U-turn (circle and square turn).

The first stage is to decide whether ILG will be performed at home or at the laboratory or hospital (**Figure 3**). Among the 78 studies, 67 were set in a laboratory or hospital only, 9 assessed gait only when the patient stayed at home and 2 studies were performed in both environments.

##### Floor type and sequence of steps

The second stage involves floor type and the exact sequence of steps the patient is asked to perform, from the most basic to the most elaborate (**Figure 3**). The main characteristics included short sequences (<20 m in total) on an unlevelled surface, without a U-turn or sit-to-stand transition, with speed left to the convenience of the participant.

##### Speed

Walking speed is an additional parameter that can be tuned. Three main solutions were envisioned: letting the participant choose the speed deemed the most convenient (“self-selected speed”), instructing the participant to walk as fast as possible (“fastest speed walk”) or imposing a given speed as defined by the literature. Overall, 63 of the 78 articles did not justify their choice and 15 examined the issue; 9 of the 15 studies repeated the protocol under several speeds (from 2 to 6 speeds) to describe only the effect of speed on parameters or to perform further analysis: by subgroup analysis, linear mixed model with speed as a fixed effect or a dependent variable, comparison of parameters normalized to interpolated speed. The 6 other studies explored the effect of self-selected speed, by using correlation of parameters to speed, subgroup analysis or two-way mixed effects analysis of covariance with speed as a covariate. Overall, numerous parameters highly varied with speed.

##### Sensitization tactics: the use of specific conditions

The fourth step in the design of the protocol concerned the use of specific sensitizing conditions. Studies included dual-task (Hsu et al., 2014; Howell et al., 2015; Gillain et al., 2016; Henderson et al., 2016; Jaywant et al., 2016), eyes-closed walk, narrow-step width, and obstacle negotiation. Except for 4 studies, all concerned neurological patients.

##### Aid

Finally, patients could be asked to walk with their usual mobility aid (walker, cane, crutches or braces). In the absence of any details in the articles reviewed, we assumed that patients walked without any help.

#### Choice and Position of the Sensor

**FIGURE 4:**
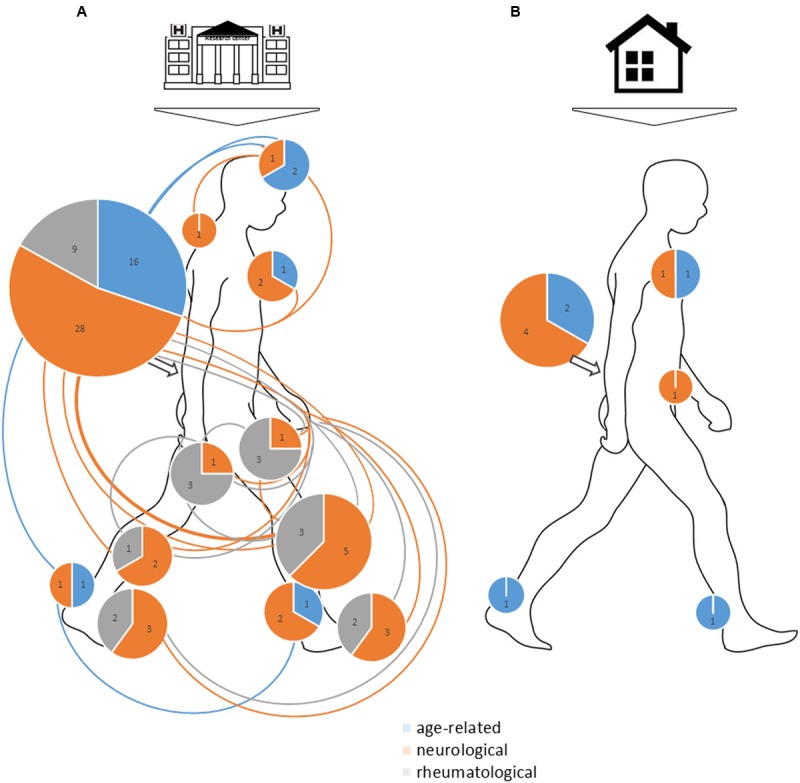
**Overview of sensor position in (A)** laboratory or clinical settings and **(B)** ambulatory settings. The size of the circles represents the total number of studies (neurological, age-related, rheumatological, others). Numbers in the circles represent the number of studies for each of these four specialties. For sensor position, one line joining several body parts represents one study using sensors attached to these body parts. When sensor was attached to a foot, ankle or thigh unilaterally, we added one study point on the right side and none on the left side.

**FIGURE 5:**
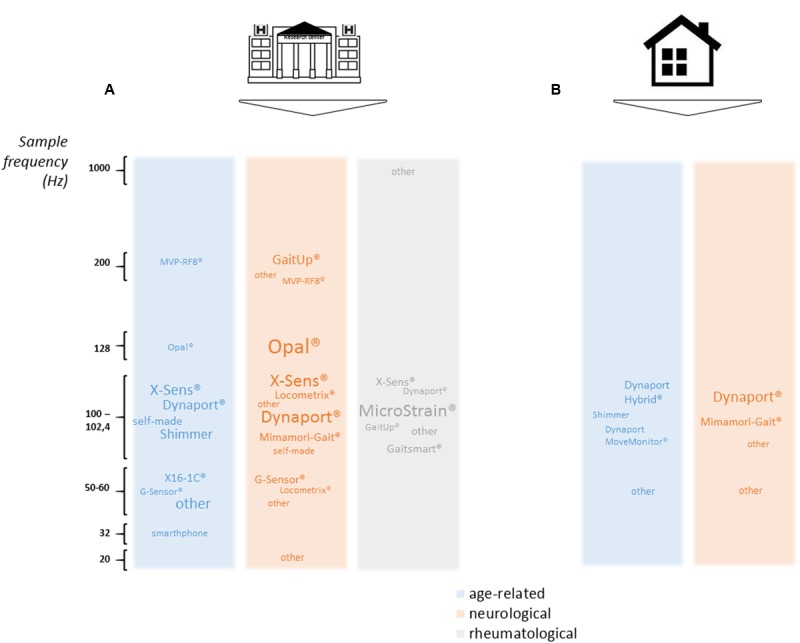
**Overview of main sensors used as a function of frequencies in (A)** laboratory or clinical settings and **(B)** ambulatory settings.

Inertial sensors were not all the same (**Figures 4, 5**). Several specifications are also taken into account: sampling frequency and low position drift, of major importance for precision of data; size and weight, with increasing size and weight potentially affecting gait; battery if ambulatory measures are being performed; cost; sensitivity to magnetic fields, which can be an issue when used with robotic orthoses for instance; availability of raw unfiltered data; and software, the possibility to synchronize with other sensors such as video motion analysis for validation and electromyography for muscle activity analysis.

The selected articles mainly used 100–200 Hz sampling frequency, with neurology studies more prone to using 200-Hz sampling frequency than other types of studies, which reflects differences in types of parameters computed (more dynamic parameters than in rheumatology studies). Many sensors were tested (see **Figure 5** for names and sampling frequency used), but 2 stand out: Dynaport^®^ Hybrid (50% of ambulatory measures, 11% of laboratory or clinical measures) and XSens^®^ MtW (14% of laboratory or clinical measures).

Overall, 16% of all studies reviewed recorded from more than one IMU, with a maximum of 7 sensors. Half were neurology studies (26% of neurology studies). None involved ambulatory analysis. All setups are represented in **Figure 4** for both laboratory or clinical and ambulatory settings. The most widely chosen position was the lower back with sensors (13% of studies in laboratory or clinical settings, of which 67% were neurological studies) or without other sensors (63% of studies in laboratory or clinical settings and 64% of ambulatory studies, 49% and 57% were neurology studies). For laboratory or clinical settings, other positions included head (4% of all studies, 3% of neurology studies only), sternum (4% of all studies, 6% of neurology studies only), upper back (4% of all studies, 3% of neurological studies), pelvis (1% of all studies, 3% of neurology studies only), bilateral thighs (6% of all studies, 2% of neurology studies only), unilateral or bilateral shanks (12% of all studies, 14% of neurology studies only), unilateral or bilateral ankles (3% of all studies, 6% of neurology studies only) and bilateral feet (7% of all studies, 9% of neurology studies only). For ambulatory studies, in addition to the lower back, placements were sternum (18% of all studies, 17% of neurology studies only), pelvis (9% of all studies, 17% of neurology studies only) and bilateral feet (9% of all studies, no neurology studies).

#### Analysis

##### Walking bout, turning and step detection

**FIGURE 6:**
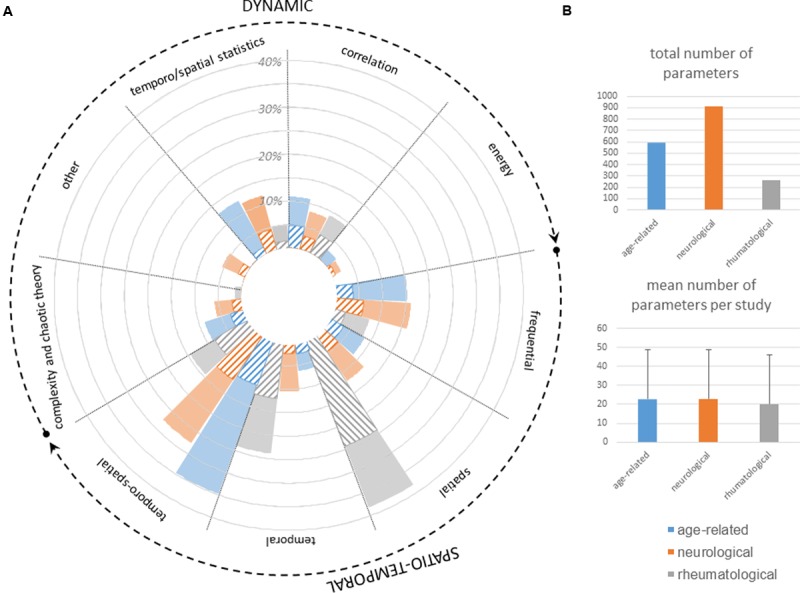
**Analysis used to compute gait features of neurologic patients as well as rheumatologic and gerontologic patients for comparison. (A)** Fully colored slices represent the percentage of total number of parameters computed in studies from the specialty (neurology, rheumatology, age-related) by using the method specified on top of the slice. Striped slices represent the percentage of parameters computed in studies from the specialty (neurology, rheumatology, age-related) that could discriminate between two cohorts in at least one study. **(B)** Total number of parameters (including all studies) and mean number per study computed in studies from the specialty (neurology, rheumatology, age-related).

One of the main remaining challenges in the quantification of gait measures using inertial sensors is robust and accurate walking-bout automatic detection (for ambulatory measurements), turning, and automatic-step detection. Signals are now precisely understood and described, which allows for manual detection (Taborri et al., 2016). Nevertheless, this process can be long and painstaking and prevents any online analysis (**Figure 6**).

Apart from one study (data not available), all ambulatory studies reviewed used automatic walking-bout detection: 45% relied on an algorithm implemented by Weiss et al. (2011), which uses a signal magnitude area (SMA) threshold combined with a frequency based filter. Other algorithms were from previously published articles by Lyons et al. (2005), Dijkstra et al. (2010), Fraccaro et al. (2014) or Yoneyama et al. (2014) or were developed by the authors by using cranio-caudal acceleration peaks and wavelet decomposition.

In studies that included a U-turn, only 3 specifically discriminated a U-turn from straight-line walking. They each used a different algorithm: one adapted from Salarian et al. (2009), one from Weiss et al. (2011) and the last developed by the authors themselves.

Last but not least, automatic step detection requires intense computation. Overall, 65% of studies included involved automatic detection with only IMUs, whereas 3% were manual, 13% relied on other sensors (video motion analysis, photoelectric cells, force plates, pressure sensors) and 19% did not specify any method. Automatic detection involved use of industrial software (BTS G-STUDIO^®^ and Locometrix^®^ in 3 studies each, and Poseidon^®^, Mobility Lab^®^ and EMG Works^®^ in 1 study each), self-designed algorithms (9 studies) or previously described models and algorithms (30 studies). At least 2 studies used several algorithms. In total, 6 studies used the inverted pendulum model-based algorithm developed by Zijlstra and Hof (2003), which has been found accurate for non-disabled adults and children as well as osteoarthritis patients and which was tested on various pathologies. The zero-crossing method developed by González et al. (2010) was used in 6 other studies, the Weiss et al. (2011) SMA threshold-based filter combined with a frequency-based filter in 5 studies, the Greene et al. (2010) algorithm in 3, and the Jasiewicz et al. (2006), Mariani et al. (2013) and Yoneyama et al. (2014) algorithms in 2 studies each. Other algorithms were developed by Fraccaro et al. (2014) and van Schooten et al. (2015).

##### Steps included in the analysis

The sequence of steps to be included in the analysis also varied largely between studies. Specifically, a decision has to be made on whether the first and last steps should be included in the analysis. Most studies (48 of 78) did not specify if any step was removed. One study kept all detected steps (Zakaria et al., 2015). Other articles removed the first meters (4 studies removed 1 m, 1 study removed 1.5 m, 5 studies removed 2 m, 2 studies removed 2.5 m, 1 study removed 3 m and 1 last study removed 3.4 m), the first steps (2 studies removed the first step, 1 other the first 3 steps and 2 others the first 4 steps) or the first seconds (5 and 30 s, respectively, for the 2 studies that chose this solution). One study analyzed only one typical stride (Rahman et al., 2015). Eventually, the last 8 studies first defined the data points to be included (between 6 and 10 middle steps and between 6 and 30 s depending on the studies) and removed the rest.

### Features

The multiplicity of parameters derived from these measurements can be highly confusing for practitioners: among the 78 studies, 453 parameters were computed, with only 102 assessed in at least 2 studies.

Computation methods ranged from the most basic to the most elaborate. Classically, gait parameters were divided into (1) spatiotemporal features, expressed as means over several steps, which reflect a typical cycle, and (2) dynamic features, which mirror the irregularity of gait for which standard deviations or coefficients of variation across several strides have been successfully complemented by more intricate extraction methods borrowing from frequential and wavelet analysis, analysis of complexity, chaos theory or fractals.

As can be appreciated in **Figure 6**, neurology and age-related studies both used the whole range of possible methods, whereas rheumatological conditions were mostly described with more straightforward spatial and temporal parameters.

## Discussion

### Protocols of Gait Evaluation

We conducted a thorough review of the literature to identify the most commonly used protocols for ILG for its use in neurology clinical practice. Not surprisingly, we found a plethora of different set-ups and tools, and the variations involved the environment, floor, instruction for speed, sensors, specific conditions and constraint and parameters assessed.

In terms of the environment, measures could be performed in ambulatory or laboratory set-ups. Ambulatory assessment is more representative of people’s gait. Indeed, the environment with ILG is physiological; hours of walking at different times of day can be recorded, which is key to valid interpretation of pattern and rhythm variability parameters that assess gait at a macro-level (Robles-García et al., 2015); and the putative “white-coat” syndrome (Andrzejewski et al., 2016) is avoided. Nevertheless, ambulatory data extraction and analysis entail great challenges because various algorithms have been developed for controlled testing, and their validity in this uncontrolled environment is questioned. Reviewed studies were mostly set up in a laboratory or clinical space (69 of 78 studies). Two studies, analyzing both ambulatory and laboratory data (**Figures 2, 3**), brought more arguments for higher sensitivity of ambulatory measures while enhancing the difficulty in controlling features that highly depend on bout length and algorithms for gait detection (Andrzejewski et al., 2016; Del Din et al., 2016a).

In terms of floor type and sequence of steps, treadmills were preferred to over-ground walking because they are space-efficient and allow for assessment at specific walking speeds. However, they are less ecological and may provide external cues, thus acting as confounders (Kang and Dingwell, 2008; Bruijn et al., 2009; Bryant et al., 2011). Long-distance or minute-long walks may be upheld because they are needed for computation of some factors (detrended fluctuation analysis, chaos theory analysis) and because they allow for assessment of fatigue and give time for patients to become familiar with the task. Nonetheless, they are limited by lack of space, time or patient capabilities.

The speed the participant should take has a strong effect on the general quality of the walk (Frenkel-Toledo et al., 2005; Beauchet et al., 2009). In the panel of articles, 3 main solutions were envisioned: letting the participant choose the speed deemed the most convenient (“self-selected speed”), instructing the participant to walk as fast as possible (“fastest speed walk”) or imposing a given speed. The first case is more physiological and preserves the walking pattern. In turn, any variable correlated with speed will be affected and conclusions should be drawn carefully. The choice of the fastest speed, which is often used in the TUG test or to evaluate a time threshold to complete the test, assesses the participant’s adaptability and sensitizes the risk of fall. Nevertheless, the pattern of walk is less physiological (Middleton et al., 2015) and performance, not quality, is evaluated. In addition, the test does not allow for exploration of speed alteration itself, which is known to bring key information (Toebes et al., 2015). Eventually, imposing a given speed allows for matching with controls, with data from the literature (Bragge et al., 2014) or between baseline and follow-up measurements (Ellis et al., 2015; Schmitz-Hubsch et al., 2016). However, as for fastest speed walking, the pattern of walk is modified and speed alteration itself is not explored. Because studies included mainly asked subjects to walk at self-selected speed only (83% of studies), speed decorrelation analysis should be performed so that the parameter group difference already accounted for by speed can be removed. Another solution would be to implement principal component analysis or sophisticated selection features analysis, to have independent parameters, which often leads to opacity in clinical interpretation.

The possibility to walk with aid is another parameter that can influence results, although it was not mentioned in the 78 studies. Patients are more likely to reproduce their usual walk when using their habitual aid, but this can be a major confounder.

As shown in **Figure 5**, some types of sensors were mainly used in the past years, among which Dynaport^®^ and XSens^®^ stood out. Some arguments for choosing Dynaport^®^ might be that it has been validated to detect walking bouts as well as steps and it has been registered as a Class I Medical Device with the US Food and Drug Administration and the European Medicines Agency, so validation of clinical trials is easier. Choosing XSens^®^ MtW could be explained in part by the availability of raw data, the possibility of easily synchronizing XSens^®^ IMUs with other XSens^®^ sensors and the option to use already-computed algorithms to change the frame of reference.

The choice of the number and exact position of sensors should consider expected outcomes (e.g., exploration of knee motion should include both thigh and distal shank IMUs), practicality (patients should feel comfortable), time to set up and ease in reproducing attachment.

Walking detection and segmentation is a main issue in gait analysis. Many different algorithms for walking bout detection have been developed and good results have been obtained with threshold-based methods (Weiss et al., 2011), zero-crossing methods (González et al., 2010), and model-based methods (Zijlstra and Hof, 2003). Because turning steps are different from straight-line walking and therefore prone to spoiling straight-line data and because they may bring insight into pathological gaits, characterization of turning steps inside the walk is of key importance. Several algorithms also exist that should be further tested.

Steps that should be included in the analysis highly depend on protocols, the environment (laboratory or ambulatory) and features that need more or fewer data points. Nevertheless, some questions should not be left unanswered because they have a strong impact on computations. Particularly, assessing steady-state walking requires a clear definition of the transition period. For frail older adults, Lindemann et al. (2008) advocate the exclusion of the first 2.5 m to be confident about assessing a purely steady-state walk.

### Semiology of Walking

The diversity of parameters is an obstacle for a comprehensive overview of pathological gaits and can be sometimes disturbing (Bloem et al., 2016). Inconsistent and uninformative interpretation of outcomes often precludes a prescriptive attitude toward the patient. After homogenization of directly related parameters, 57 parameters were extracted from the 102 parameters and classified according to seven criteria classically assessed in neurology, physical medicine and rehabilitation, gerontology and rheumatology:

– Springiness: criterion relative to gait rhythmic pattern, which includes step time, percentage time of the different step phases;
– Sturdiness: criterion relative to gait amplitude, which includes step length, range of motion of any articulation, average and maximum acceleration or speed of the foot, vertical acceleration of the lower back;
– Smoothness: criterion relative to continuousness or non-intermittency of walking, which is by definition independent of its amplitude or rhythm (Lord et al., 2013), and includes mean and maximum anteroposterior acceleration of the lower back, maximum acceleration or speed of all body parts;
– Stability: criterion relative to postural balance, which includes mediolateral range of motion of the lower back, Lyapunov exponent of the lower back in all directions, entropy;
– Steadiness: criterion relative to step regularity, which includes variation coefficients and autocorrelation coefficients of all parts and all directions;
– Symmetry: criterion relative to right/left concordance, which includes harmonic ratio, symmetry of autocorrelation coefficient, right/left symmetry of parameters of springiness and sturdiness;
– Synchronization: criterion relative to inter limbs (lower limbs and upper limbs) coordination, which includes double stance time, phase coordination index.

Speed was not considered a quality index but was left as a performance index as proposed by Lord et al. (2013). For each of the 57 parameters, the discrimination power (dP) was defined as the percentage of analyses in which the parameter was significant to the number of times the parameter was assessed (Supplementary Table S3).

Methods for automatic analysis of ILG during clinical time should be more available so that gait is measured as a follow-up. Being more systematic and understanding the semiology of ILG would allow for better and the most personalized care because each patient often requires a different rehabilitation intervention depending on their primary functional constraints.

### Limitations

A number of limitations should be considered when interpreting the results of this review. First, the dates for inclusion were limited to the past 3 years. This restriction allowed us to extract precise information from the articles, but it might have favored more recent set-ups and specially more recent sensor types over others. Second, inclusion criteria were limited to studies overtly designed to test IMUs as a tool for pathological gait assessment. Therefore, other protocols that could have been useful in healthy participants were not included. As well, the results of the quality assessment included in this review were based on the assessors’ own interpretation. We aimed to reduce subjectivity by analyzing each article 3 times, in 3 different orders and by 3 different assessors blinded to the scores given by the 2 others. Yet, subjectivity should be kept in mind.

### Prospects and Recommendations

Our review highlights the great prospects for use of IMUs in neurological practice. Nevertheless, it also emphasizes the lack of standardization and the heterogeneity of results that do not facilitate the adoption of IMUs for gait analyses.

Indeed, homogenization of protocols is needed to validate normative values and to be able to compare diseases, which is key to envisioning the use of IMUs as a tool for assessing gait quality in routine practice. Reasons for deciding on one protocol or another are not often specified in studies because they may depend on very pragmatic external factors (sensor price, laboratory or clinical space). However, this feedback is of great importance ensure that any recommendation could be followed by practitioners.

From available data, we can propose the following recommendations:

– Environment: both hospital and ambulatory assessments should be conducted depending on the pathology and the objective. Elderly fallers should be assessed in their home environment to account for external fall risk factors. Likely, patients or patients’ relatives reporting fluctuant alterations of gait during the day should be an indication for ambulatory assessments;
– Protocol: we recommend the use of 2 set-ups in the hospital. The TUG test does not appear long enough to assess gait quality. Likely, the 25-foot walk test does not include a U-turn. A short walk 10–50 m long with a U-turn in the middle should advantageously overcome this issue. The 6-min walk test would allow for assessment of fatigability and is therefore also recommended;
– Walking bout detection, walk segmentation, step detection: previously described algorithms should be made available to be tested and discussed openly before any recommendation is made.
– Features: the set of 57 parameters presented here should be informative for most diseases. It should be tested on several set-ups to validate reliability and several diseases and healthy participants should be assessed so as to remove redundancy and provide normative values. We recommend the use of the 7 clinical criteria that could provide relevant feedback to both the patient and the clinician.

These recommendations are preliminary and should be challenged by larger studies of several diseases. Stronger and more precise recommendations could then be agreed on by clinicians and regulatory societies. Their implementation would then require the development of an integrated and comprehensive environment for wide-scale adoption by clinicians. This enterprise requires involvement and strengthened communication among all fundamental actors in the process (Maetzler et al., 2016). Upstream work on sensors is expected from industry and engineers, as is further study and testing by researchers to investigate potential confounders including patient morphology and the protocol testing method and to develop markers for diagnosis, disease severity, prognosis, fall risk, and treatment efficiency. The need for more robust gait detection algorithms — tested with patients early in the process — and computation of clinically relevant features emphasizes the centrality of the computer scientist (Kubota et al., 2016). This need would benefit from recent development of computer-aided diagnosis methods including machine learning. Eventually, feedback from the patient should be welcomed and sought at every stage. Only then will new applications be delineated and the contribution of ILG to improving gait analysis and understanding gait disorders be fully appreciated.

## Author Contributions

AV: design, quality review, main author. RB: quality review and review. SB: review. DR: design, quality review and review. P-PV: review.

## Conflict of Interest Statement

The authors declare that the research was conducted in the absence of any commercial or financial relationships that could be construed as a potential conflict of interest.
