# Supplementary material for: Inertial Sensors to Assess Gait Quality in Patients with Neurological Disorders: A Systematic Review of Technical and Analytical Challenges
Source: Front Psychol. 2017 May 18;8:817. doi: 10.3389/fpsyg.2017.00817 (PMC5435996; doi:10.3389/fpsyg.2017.00817)
Supplement: Supplementary file 1 [file Data_Sheet_1.docx]

Supplementary Material

Inertial sensors to assess gait quality in patients with neurological disorders: a systematic review

Aliénor VIENNE* MSc, Rémi BARROIS MSc, Stéphane BUFFAT MD PhD, Damien RICARD MD PhD and Pierre Paul VIDAL MD PhD

*** Correspondence:** Corresponding Author: [alienor.vienne@parisdescartes.fr](mailto:alienor.vienne@parisdescartes.fr)

1. **20-item quality checklist**

*Reporting*

1. Is the hypothesis/aim/objective of the study clearly described? (1 point)
2. Are the main outcomes to be measured clearly described in the Introduction or Methods section? (1 point)

If the main outcomes are first mentioned in the Results, the score should be 0.

1. Is the protocol clearly described? (2 points).

Description should include the eight following elements: ambulatory or distance or time of walk, type of floor (ambulatory, treadmill, unlevelled, slop), sequence of steps (ambulatory or U-turn), sequence of steps (ambulatory or sit-to-stand transition), sensor type, sensor frequency, sensor position, speed (self-selected or imposed). Give 0 point if two elements or more are missing, 1 point if one element is missing, 2 points if the eight elements are specified.

1. Are walking bout detection, walking segment detection and step detection methods clearly described or referred to? (1 point)

Walk detection refers to detection of walking bouts (required for ambulatory studies or when parameters are computed on the whole signal but the whole time of walk is included in the processing). Segment detection includes detection of: transition (acceleration and deceleration phases and U-turn when they are not included in the analyzed steps). Step detection includes detection of all steps. Description should allow one to answer the three following questions: is the method automatic or manual? Which signal is analyzed (IMU or another sensor)? If it relies only on IMU, which axis and which peak is used? When needed, the three elements should be either explained in the method or referred to an article where explanation allow answers to questions above mentionned. Give 0 point if two elements or more are not well described, ½ point if one element is not well described, 1 point if all elements are well described. If neither of the three elements are needed (eg laboratory gait analysis were first 5 and last 5 sec are discarded and parameters are computed on the whole signal), the item is not quoted.

1. Are the number of trials and steps included in the analysis clearly specified? (1 point)
2. Are the characteristics of the patients included in the study clearly described? (1 point).

In cohort studies and trials, inclusion and/or exclusion criteria should be given. In case-control studies, a case-definition and the source for controls should be given.

1. Are the distributions of principal confounders in each group of subjects to be compared clearly described? (2 points)

A list should be given.

1. Are the main findings of the study clearly described? (1 point)

When they exist, quantitative findings should be reported in the ’results’ section and discussed in the ’discussion’ section for the main findings.

1. Does the study provide estimates of the random variability in the data for the main outcomes? (1 point)

In non-normally distributed data the inter-quartile range of results should be reported. In normally distributed data the standard error, standard deviation or confidence intervals should be reported. If the distribution of the data is not described it must be assumed that the estimates used were appropriate and the item should be scored 1.

1. Have actual probability values been reported (e.g. 0.035 rather than <0.05) for the main outcomes (when significant) except where the probability value is less than 0.001%? (1 point)

When no result is significant, the item is not quoted.

*External validity*

1. Were the subjects asked to participate in the study representative of the entire population from which they were recruited? (1 point)

The study must identify the source population for patients and describe how the patients were selected. Patients would be representative if they comprised the entire source population, an unselected sample of consecutive patients, or a random sample. Random sampling is only feasible where a list of all members of the relevant population exists. Where a study does not report the proportion of the source populations from which the patients are derived, the score should be 0 (unable to determine).

1. Were those subjects who were prepared to participate representative of the entire population from which they were recruited? (2 points)

The proportion of those asked who agreed should be stated. Validation that the sample was representative would include demonstrating that the distribution of the main confounding factors was the same in the study sample and the source population.

1. Was there validation of the sensor used? (1 point)

If the sensor is self-made and no analysis of reliability has been made in either the article or a precedent one, the score should be 0.

*Internal validity - Bias*

1. If any of the results of the study were based on ‘data dredging’, was this made clear? (1 point)

Any analyses that had not been planned at the outset of the study should be clearly indicated. If no retrospective unplanned subgroup analyses were reported, then score 1.

1. Were the statistical tests used to assess the main outcomes appropriate? (1 point)

If no test for normality or no post-hoc corrections has been made when needed, only 1/2 point should be given. If both criteria fail, score should be 0.

1. Were the main outcome measures used accurate (valid and reliable)? (1 point)

For studies were the outcome measures are clearly described, the score should be 1. For studies which refer to other work or that demonstrates the outcome measures are accurate, the score should be 1.

*Internal validity - Confounding (Selection Bias)*

1. Were the patients in different intervention groups (trials and cohort studies) or were the cases and controls (case-control studies) recruited from the same population? (1 point)

Patients for all comparison groups should be selected from the same hospital(s) or institution(s). Patients and controls should not have significantly different age. No point should be given whe no information can be found regarding the source of patients included.

1. Were study subjects in different intervention groups (trials and cohort studies) or were the cases and controls (case-control studies) recruited over the same period of time? (1 point)
2. Was there adequate adjustment for confounding in the analyses from which the main findings were drawn? (1 point)

This question should be scored 0 for trials if the main conclusions of the study were; i) based on analyses of treatment rather than intention to treat; ii) the distribution of known confounders in the different treatment groups was not described; or iii) the distribution of known confounders differed between the treatment groups but was not taken into account in the analyses. In nonrandomized studies if the effect of the main confounders was not investigated or confounding was demonstrated but no adjustment was made in the final analyses the question should be scored 0.

*Power*

1. Did the study have sufficient power to detect a clinically important effect where the probability value for a difference being due to chance is less than 5%? (5 points)
2. **Results - raw data**

Neurology studies

| item | 1 | 2 | 3 | 4 | 5 | 6 | 7 | 8 | 9 | 10 | 11 | 12 | 13 | 14 | 15 | 16 | 17 | 18 | 19 | 20 |  |  |  |  |  |
| --- | --- | --- | --- | --- | --- | --- | --- | --- | --- | --- | --- | --- | --- | --- | --- | --- | --- | --- | --- | --- | --- | --- | --- | --- | --- |
| maximum number of points | 1 | 1 | 2 | 1 | 1 | 1 | 1 | 1 | 1 | 1 | 1 | 2 | 1 | 1 | 1 | 1 | 1 | 1 | 1 | 5 | **REPORTING** | **EXTERNAL** | **INTERNAL** | **POWER** | **TOTAL** |
| Andrzejewski et al. 2016 | 1 | 1 | 2 | 0 | 0 | 0 | 0 | 1 | 1 | 0 | 0 | 0 | 1 | 0 | 0,5 | 1 | 0 | 0 | 0 | 0 | 33% | 55% | 25% | 25% | **0%** |
| Bregou Bourgeois et al. 2014 | 1 | 1 | 2 | 1 | 0 | 1 | 1 | 1 | 1 | 1 | 0 | 0 | 1 | 1 | 0,5 | 1 | 0 | 0 | 0 | 0 | 91% | 25% | 42% | 0% | **52%** |
| Brodie, Canning, et al. 2015 | 1 | 1 | 2 | 0 | 1 | 1 | 1 | 1 | 1 | 1 | 0 | 0 | 1 | 1 | 0,5 | 1 | 0 | 0 | 0 | 0 | 91% | 25% | 42% | 0% | **52%** |
| Chini et al. 2016 | 1 | 1 | 1 | 1 | 1 | 1 | 0 | 1 | 1 | 0 | 0 | 0 | 1 | 1 | 0 | 1 | 0 | 0 | 0 | 0 | 73% | 25% | 33% | 0% | **42%** |
| Collett et al. 2014 | 1 | 1 | 2 | 1 | 1 | 1 | 0 | 1 | 1 | 1 | 0 | 0 | 1 | 1 | 0,5 | 1 | 0 | 0 | 0 | 0 | 91% | 25% | 42% | 0% | **52%** |
| Del Din, Godfrey, and Rochester 2015 | 1 | 1 | 2 | 0 | 1 | 1 | 1 | 1 | 1 | 1 | 0 | 0 | 1 | 1 | 0,5 | 1 | 0 | 0 | 1 | 0 | 91% | 25% | 58% | 0% | **56%** |
| Del Din et al. 2016 | 1 | 1 | 2 | 0,5 | 0 | 1 | 0 | 1 | 1 | 1 | 0 | 0 | 1 | 0 | 0,5 | 1 | 0 | 0 | 0 | 0 | 77% | 25% | 25% | 0% | **42%** |
| Demonceau et al. 2015 | 1 | 1 | 2 | 1 | 1 | 1 | 1 | 1 | 0 | 0 | 0 | 0 | 1 | 1 | 1 | 1 | 0 | 0 | 1 | 0 | 82% | 25% | 67% | 0% | **54%** |
| Doi et al. 2015 | 1 | 1 | 2 | 1 | 1 | 1 | 1 | 1 | 1 | 1 | 0 | 0 | 1 | 1 | 0 | 1 | 1 | 1 | 1 | 0 | 100% | 25% | 83% | 0% | **65%** |
| Ellis et al. 2015 | 1 | 1 | 2 | 1 | 0 | 1 | 1 | 1 | 1 | 0 | 0 | 0 | 1 | 1 | 0 | 1 | 1 | 0 | 0 | 0 | 82% | 25% | 50% | 0% | **50%** |
| Gillain et al. 2016 | 1 | 1 | 2 | 1 | 1 | 1 | 1 | 1 | 1 | 1 | 0 | 0 | 1 | 1 | 0,5 | 1 | 1 | 1 | 0 | 0 | 100% | 25% | 75% | 0% | **63%** |
| Hatanaka et al. 2016 | 1 | 1 | 2 | 1 | 0 | 1 | 1 | 1 | 1 | 0 | 0 | 0 | 1 | 1 | 1 | 1 | 0 | 0 | 0 | 0 | 82% | 25% | 50% | 0% | **50%** |
| Henderson et al. 2016 | 1 | 1 | 1 | 0,5 | 1 | 1 | 1 | 1 | 1 | 1 | 1 | 2 | 1 | 1 | 0,5 | 1 | 1 | 0 | 1 | 1 | 86% | 100% | 75% | 20% | **73%** |
| Howell, Osternig, and Chou 2015 | 1 | 1 | 2 | 1 | 0 | 1 | 1 | 1 | 1 | 1 | 0 | 0 | 1 | 1 | 1 | 1 | 0 | 0 | 0 | 0 | 91% | 25% | 50% | 0% | **54%** |
| Hsu et al. 2014 | 1 | 1 | 2 | 1 | 0 | 1 | 1 | 1 | 1 | 1 | 0 | 0 | 0 | 1 | 0 | 1 | 0 | 0 | 0 | 0 | 91% | 0% | 33% | 0% | **46%** |
| Jaywant et al. 2016 | 1 | 1 | 2 | 1 | 0 | 1 | 1 | 1 | 1 | 0 | 1 | 2 | 1 | 0 | 0 | 1 | 1 | 1 | 0 | 5 | 82% | 100% | 50% | 100% | **81%** |
| Kleiner et al. 2015 | 1 | 1 | 2 | 0,5 | 0 | 1 | 1 | 1 | 1 | 0 | 0 | 0 | 1 | 1 | 0,5 | 1 | 0 | 0 | 0 | 0 | 77% | 25% | 42% | 0% | **46%** |
| Lauretani et al. 2016* | 1 | 1 | 2 | 0 | 0 | 1 | - | 1 | 0 | 0 | 0 | 0 | 1 | 0 | 0 | 1 | - | - | - | 0 | 60% | 25% | 33% | 0% | **36%** |
| Mancini et al. 2016 | 1 | 1 | 2 | 1 | 1 | 0 | 0 | 1 | 1 | 1 | 0 | 0 | 1 | 0 | 0 | 1 | 0 | 0 | 0 | 0 | 82% | 25% | 17% | 0% | **42%** |
| Martinez-Ramirez et al. 2016 | 1 | 1 | 2 | 1 | 1 | 1 | 1 | 1 | 1 | 1 | 0 | 0 | 1 | 1 | 1 | 1 | 0 | 1 | 0 | 0 | 100% | 25% | 67% | 0% | **62%** |
| Martino Cinnera et al. 2015* | 1 | 1 | 2 | 1 | 1 | 1 | - | 1 | 1 | - | 0 | 0 | 1 | 0 | 0 | 1 | - | - | - | 0 | 100% | 25% | 33% | 0% | **52%** |
| Matsushima et al. 2015 | 1 | 1 | 2 | 0 | 1 | 0 | 1 | 1 | 1 | 0 | 0 | 1 | 0 | 1 | 0,5 | 1 | 0 | 0 | 0 | 0 | 73% | 25% | 42% | 0% | **44%** |
| Mirelman et al. 2014 | 1 | 1 | 2 | 1 | 0 | 1 | 0 | 1 | 1 | 1 | 1 | 1 | 1 | 0 | 1 | 1 | 1 | 1 | 1 | 0 | 82% | 75% | 83% | 0% | **65%** |
| Motta et al. 2016 | 1 | 1 | 2 | 0 | 0 | 1 | 0 | 1 | 1 | 1 | 0 | 0 | 1 | 1 | 0,5 | 1 | 0 | 0 | 0 | 1 | 48% | 73% | 25% | 42% | **20%** |
| Pan et al. 2015 | 1 | 1 | 2 | 1 | 0 | 1 | 1 | 1 | 1 | 1 | 0 | 0 | 1 | 0 | 0,5 | 1 | 1 | 1 | 0 | 0 | 91% | 25% | 58% | 0% | **56%** |
| Perrochon et al. 2015 | 1 | 1 | 2 | 1 | 0 | 1 | 1 | 1 | 1 | 0 | 1 | 0 | 1 | 1 | 0,5 | 1 | 1 | 1 | 0 | 0 | 82% | 50% | 75% | 0% | **60%** |
| Reynard et al. 2014 | 1 | 1 | 2 | 1 | 1 | 1 | 1 | 1 | 1 | 1 | 1 | 1 | 1 | 1 | 0,5 | 1 | 1 | 1 | 1 | 4 | 100% | 75% | 92% | 80% | **90%** |
| Saether et al. 2014 | 1 | 1 | 2 | 1 | 1 | 1 | 1 | 1 | 1 | 1 | 0 | 0 | 1 | 1 | 0 | 1 | 0 | 0 | 1 | 0 | 100% | 25% | 50% | 0% | **58%** |
| Saether et al. 2015 | 1 | 1 | 2 | 0 | 0 | 1 | 1 | 1 | 1 | 1 | 1 | 0 | 0 | 0 | 0,5 | 1 | 0 | 0 | 1 | 0 | 82% | 25% | 42% | 0% | **48%** |
| Schmitz-Hubsch et al. 2016 | 1 | 1 | 2 | 1 | 0 | 1 | 1 | 1 | 1 | 1 | 0 | 0 | 1 | 1 | 0 | 1 | 0 | 0 | 0 | 0 | 91% | 25% | 33% | 0% | **50%** |
| Sejdic et al. 2014 | 1 | 1 | 2 | 1 | 0 | 1 | 0 | 1 | 1 | 0 | 0 | 0 | 1 | 1 | 0,5 | 1 | 0 | 0 | 0 | 0 | 73% | 25% | 42% | 0% | **44%** |
| Shirai et al. 2015 | 1 | 1 | 2 | 0 | 0 | 0 | 0 | 1 | 1 | 1 | 0 | 0 | 1 | 1 | 0,5 | 1 | 0 | 0 | 0 | 2 | 64% | 25% | 42% | 40% | **48%** |
| Summa et al. 2016 | 1 | 1 | 2 | 1 | 0 | 1 | 0 | 0 | 0 | 0 | 0 | 0 | 1 | 1 | 0,5 | 1 | 0 | 0 | 1 | 0 | 55% | 25% | 58% | 0% | **40%** |
| Weiss et al. 2014 | 1 | 1 | 2 | 1 | 0 | 1 | 1 | 1 | 1 | 1 | 0 | 0 | 1 | 1 | 0,5 | 1 | 1 | 1 | 0 | 0 | 91% | 25% | 75% | 0% | **60%** |
| Weiss et al. 2015a | 1 | 1 | 2 | 1 | 1 | 1 | 1 | 1 | 1 | 1 | 1 | 1 | 1 | 1 | 1 | 1 | 1 | 1 | 1 | 0 | 100% | 75% | 100% | 0% | **77%** |
| Weiss et al. 2015b | 1 | 1 | 2 | 1 | 0 | 1 | 1 | 1 | 1 | 1 | 0 | 0 | 1 | 1 | 1 | 1 | 1 | 1 | 0 | 0 | 91% | 25% | 83% | 0% | **62%** |
| Yoneyama et al. 2015 | 1 | 1 | 2 | 1 | 0 | 1 | 1 | 1 | 1 | 0 | 0 | 0 | 1 | 1 | 1 | 1 | 0 | 0 | 0 | 0 | 82% | 25% | 50% | 0% | **50%** |
| Zollinger et al. 2016 | 1 | 1 | 2 | 1 | 0 | 1 | 1 | 1 | 1 | 0 | 0 | 0 | 1 | 1 | 0 | 1 | 0 | 0 | 0 | 0 | 82% | 25% | 33% | 0% | **46%** |

Age and morphology-related studies

| item | 1 | 2 | 3 | 4 | 5 | 6 | 7 | 8 | 9 | 10 | 11 | 12 | 13 | 14 | 15 | 16 | 17 | 18 | 19 | 20 |  |  |  |  |  |
| --- | --- | --- | --- | --- | --- | --- | --- | --- | --- | --- | --- | --- | --- | --- | --- | --- | --- | --- | --- | --- | --- | --- | --- | --- | --- |
| maximum number of points | 1 | 1 | 2 | 1 | 1 | 1 | 1 | 1 | 1 | 1 | 1 | 2 | 1 | 1 | 1 | 1 | 1 | 1 | 1 | 5 | **REPORTING** | **EXTERNAL** | **INTERNAL** | **POWER** | **TOTAL** |
| Arvin et al. 2016 | 1 | 1 | 2 | 1 | 0 | 0 | 0 | 1 | 1 | 0 | 0 | 0 | 1 | 1 | 0,5 | 1 | 0 | 0 | 0 | 3 | 64% | 25% | 42% | 60% | **52%** |
| Bragge et al. 2014 | 1 | 1 | 3 | 1 | 1 | 1 | 1 | 1 | 1 | 1 | 1 | 1 | 1 | 1 | 0,5 | 1 | 1 | 1 | 0 | 0 | 109% | 75% | 75% | 0% | **75%** |
| Brodie et al. 2015a | 1 | 1 | 2 | 1 | 0 | 1 | 1 | 1 | 1 | 0 | 0 | 0 | 1 | 1 | 0 | 1 | 1 | 1 | 0 | 0 | 82% | 25% | 67% | 0% | **54%** |
| Brodie et al. 2015b | 1 | 1 | 1 | 0 | 1 | 1 | 1 | 1 | 1 | 1 | 0 | 0 | 1 | 1 | 0 | 1 | 0 | 0 | 0 | 0 | 82% | 25% | 33% | 0% | **46%** |
| Buckinx et al. 2015 | 1 | 1 | 2 | 0 | 0 | 1 | 1 | 1 | 1 | 1 | 1 | 0 | 1 | 1 | 0,5 | 1 | 1 | 1 | 1 | 0 | 82% | 50% | 92% | 0% | **63%** |
| Cui et al. 2014 | 1 | 1 | 2 | 0 | 0 | 1 | 1 | 1 | 1 | 1 | 0 | 0 | 1 | 1 | 0,5 | 1 | 1 | 1 | 1 | 1 | 82% | 25% | 92% | 20% | **63%** |
| Galan-Mercant and Cuesta-Vargas 2014 | 1 | 1 | 2 | 1 | 0 | 1 | 1 | 1 | 0 | 1 | 0 | 1 | 1 | 0 | 0,5 | 1 | 0 | 0 | 0 | 0 | 82% | 50% | 25% | 0% | **48%** |
| Greene et al. 2014 | 1 | 1 | 2 | 1 | 0 | 1 | 1 | 1 | 1 | 1 | 0 | 0 | 1 | 0 | 1 | 1 | 1 | 1 | 0 | 0 | 91% | 25% | 67% | 0% | **58%** |
| Howcroft et al. 2014 | 1 | 1 | 0 | 1 | 0 | 0 | 0 | 1 | 1 | 0 | 0 | 0 | 1 | 1 | 0,5 | 1 | 0 | 0 | 0 | 0 | 45% | 25% | 42% | 0% | **33%** |
| Howcroft et al. 2016 | 1 | 1 | 2 | 1 | 1 | 1 | 1 | 1 | 1 | 1 | 0 | 0 | 1 | 1 | 1 | 1 | 0 | 0 | 0 | 0 | 100% | 25% | 50% | 0% | **58%** |
| Ihlen, Weiss, Beck, et al. 2016 | 1 | 1 | 2 | 1 | 1 | 1 | 1 | 1 | 1 | 1 | 0 | 0 | 1 | 1 | 0,5 | 1 | 1 | 1 | 0 | 0 | 100% | 25% | 75% | 0% | **63%** |
| Ihlen, Weiss, Bourke, et al. 2016 | 1 | 1 | 2 | 1 | 0 | 1 | 1 | 1 | 1 | 0 | 0 | 0 | 1 | 1 | 1 | 1 | 1 | 1 | 0 | 0 | 82% | 25% | 83% | 0% | **58%** |
| Kobsar et al. 2014 | 1 | 1 | 2 | 1 | 1 | 1 | 0 | 1 | 1 | 0 | 0 | 0 | 0 | 1 | 0,5 | 1 | 0 | 0 | 1 | 0 | 82% | 0% | 58% | 0% | **48%** |
| Martinez-Ramirez et al. 2015 | 1 | 1 | 2 | 1 | 1 | 1 | 1 | 1 | 1 | 0 | 1 | 0 | 1 | 0 | 1 | 1 | 0 | 0 | 0 | 0 | 91% | 50% | 33% | 0% | **54%** |
| Martinikorena et al. 2016 | 1 | 1 | 2 | 1 | 1 | 1 | 1 | 1 | 1 | 0 | 0 | 0 | 1 | 0 | 0,5 | 1 | 1 | 1 | 0 | 0 | 91% | 25% | 58% | 0% | **56%** |
| Matsumoto et al. 2016 | 1 | 1 | 2 | 1 | 1 | 1 | 1 | 1 | 1 | 1 | 0 | 1 | 1 | 1 | 0,5 | 1 | 1 | 1 | 1 | 0 | 100% | 50% | 92% | 0% | **71%** |
| Pau et al. 2014 | 1 | 1 | 2 | 1 | 0 | 1 | 1 | 1 | 1 | 0 | 1 | 2 | 1 | 1 | 0 | 1 | 1 | 1 | 1 | 0 | 82% | 100% | 83% | 0% | **69%** |
| Reginatto et al. 2015* | 1 | 1 | 2 | 1 | 0 | 1 | - | 1 | 1 | - | 0 | 0 | 1 | 1 | 0 | 1 | - | - | - | 0 | 89% | 25% | 67% | 0% | **52%** |
| Rivolta et al. 2015 | 1 | 1 | 2 | 0 | 0 | 1 | 1 | 1 | 1 | 0 | 0 | 0 | 1 | 1 | 0,5 | 1 | 1 | 1 | 0 | 0 | 73% | 25% | 75% | 0% | **52%** |
| Scaglioni-Solano and Aragon-Vargas 2015 | 1 | 1 | 2 | 1 | 1 | 1 | 1 | 1 | 1 | 0 | 0 | 0 | 0 | 1 | 0,5 | 1 | 1 | 0 | 1 | 0 | 91% | 0% | 75% | 0% | **56%** |
| Sheehan et al. 2014 | 1 | 1 | 2 | 1 | 0 | 1 | 1 | 1 | 1 | 0 | 1 | 0 | 1 | 1 | 1 | 1 | 1 | 1 | 1 | 0 | 82% | 50% | 100% | 0% | **65%** |
| Shin, An, and Yoo 2015 | 1 | 1 | 2 | 1 | 1 | 1 | 1 | 1 | 1 | 0 | 0 | 0 | 1 | 1 | 0 | 1 | 1 | 1 | 0 | 0 | 91% | 25% | 67% | 0% | **58%** |
| Toebes et al. 2015 | 1 | 1 | 2 | 1 | 1 | 1 | 1 | 1 | 1 | 0 | 0 | 0 | 1 | 1 | 0,5 | 1 | 1 | 1 | 1 | 0 | 91% | 25% | 92% | 0% | **63%** |
| Valenti, Bonomi, and Westerterp 2015 | 1 | 1 | 2 | 0 | 1 | 1 | 1 | 1 | 1 | 0 | 0 | 0 | 1 | 1 | 0,5 | 1 | 1 | 1 | 1 | 0 | 82% | 25% | 92% | 0% | **60%** |
| van Schooten et al. 2016 | 1 | 1 | 2 | 1 | 0 | 1 | 1 | 1 | 0 | 0 | 1 | 1 | 1 | 1 | 0,5 | 1 | 1 | 1 | 1 | 0 | 73% | 75% | 92% | 0% | **63%** |
| Zakaria et al. 2015 | 1 | 1 | 2 | 1 | 1 | 0 | 0 | 1 | 1 | 1 | 0 | 0 | 0 | 1 | 0 | 1 | 1 | 1 | 0 | 0 | 82% | 0% | 67% | 0% | **50%** |

Rheumatology studies

| item | 1 | 2 | 3 | 4 | 5 | 6 | 7 | 8 | 9 | 10 | 11 | 12 | 13 | 14 | 15 | 16 | 17 | 18 | 19 | 20 |  |  |  |  |  |
| --- | --- | --- | --- | --- | --- | --- | --- | --- | --- | --- | --- | --- | --- | --- | --- | --- | --- | --- | --- | --- | --- | --- | --- | --- | --- |
| maximum number of points | 1 | 1 | 2 | 1 | 1 | 1 | 1 | 1 | 1 | 1 | 1 | 2 | 1 | 1 | 1 | 1 | 1 | 1 | 1 | 5 | **REPORTING** | **EXTERNAL** | **INTERNAL** | **POWER** | **TOTAL** |
| Arvin et al. 2016 | 1 | 1 | 2 | 1 | 0 | 0 | 1 | 1 | 1 | 1 | 0 | 0 | 1 | 1 | 0,5 | 1 | 1 | 1 | 0 | 0 | 82% | 25% | 75% | 0% | **56%** |
| Barrois et al. 2015 | 1 | 1 | 2 | 1 | 0 | 1 | 0 | 1 | 1 | 0 | 0 | 0 | 1 | 0 | 0,5 | 1 | 0 | 0 | 0 | 0 | 73% | 25% | 25% | 0% | **40%** |
| Bolink et al. 2015a | 1 | 1 | 2 | 1 | 0 | 1 | 1 | 1 | 1 | 1 | 1 | 0 | 1 | 1 | 0 | 1 | 0 | 0 | 0 | 0 | 91% | 50% | 33% | 0% | **54%** |
| Bolink et al. 2015b | 1 | 1 | 2 | 1 | 0 | 1 | 1 | 1 | 1 | 1 | 1 | 0 | 1 | 1 | 0,5 | 1 | 1 | 1 | 0 | 0 | 91% | 50% | 75% | 0% | **63%** |
| Bolink et al. 2016 | 1 | 1 | 1 | 1 | 0 | 1 | 1 | 1 | 1 | 1 | 1 | 1 | 1 | 1 | 0,5 | 1 | 1 | 1 | 0 | 0 | 82% | 75% | 75% | 0% | **63%** |
| Chopra et al. 2014 | 1 | 1 | 2 | 1 | 1 | 1 | 1 | 1 | 1 | 0 | 0 | 0 | 1 | 1 | 0,5 | 1 | 0 | 1 | 0 | 0 | 91% | 25% | 58% | 0% | **56%** |
| Elbaz et al. 2016 | 1 | 1 | 2 | 1 | 0 | 1 | 1 | 1 | 1 | 1 | 0 | 0 | 1 | 1 | 0,5 | 1 | 0 | 0 | 1 | 5 | 91% | 25% | 58% | 100% | **75%** |
| Henchoz et al. 2015 | 1 | 1 | 2 | 1 | 0 | 1 | 0 | 1 | 1 | 0 | 0 | 0 | 1 | 0 | 0 | 1 | 0 | 0 | 1 | 0 | 73% | 25% | 33% | 0% | **42%** |
| Hjorth et al. 2014 | 1 | 1 | 1 | 1 | 0 | 1 | 1 | 1 | 1 | 1 | 1 | 2 | 1 | 1 | 0,5 | 1 | 0 | 0 | 0 | 0 | 82% | 100% | 42% | 0% | **60%** |
| Patterson et al. 2014 | 1 | 1 | 2 | 1 | 1 | 1 | 1 | 1 | 1 | 1 | 0 | 0 | 1 | 1 | 0 | 1 | 0 | 0 | 0 | 0 | 100% | 25% | 33% | 0% | **54%** |
| Rahman et al. 2015 | 1 | 1 | 2 | 0 | 0 | 1 | 1 | 1 | 1 | 0 | 0 | 0 | 1 | 0 | 0,5 | 1 | 1 | 0 | 0 | 0 | 73% | 25% | 42% | 0% | **44%** |
| Rapp et al. 2015 | 1 | 1 | 2 | 1 | 1 | 1 | 1 | 1 | 1 | 1 | 0 | 0 | 1 | 1 | 0,5 | 1 | 0 | 0 | 1 | 0 | 100% | 25% | 58% | 0% | **60%** |
| Staab et al. 2014 | 1 | 1 | 2 | 1 | 0 | 1 | 1 | 1 | 1 | 0 | 0 | 0 | 1 | 1 | 0 | 1 | 0 | 0 | 1 | 5 | 82% | 25% | 50% | 100% | **69%** |
| Tadano et al. 2016 | 1 | 1 | 2 | 1 | 0 | 1 | 1 | 1 | 1 | 1 | 0 | 0 | 1 | 1 | 0 | 1 | 0 | 0 | 0 | 0 | 91% | 25% | 33% | 0% | **50%** |

1. **Results – Statistics**

Total statistics:

| **quality** | | **no.** | **min total** | **max total** |
| --- | --- | --- | --- | --- |
| *low* | neurological | 14 | 33% | 48% |
|  | all | 21 | 33% | 48% |
| *medium* | neurological | 19 | 50% | 65% |
|  | all | 50 | 50% | 69% |
| *high* | neurological | 4 | 73% | 81% |
|  | all | 7 | 71% | 90% |

Statistics per category:

| category  quality | | **reporting** | **external** | **internal** | **power** |
| --- | --- | --- | --- | --- | --- |
| *low* | neurological | 72% | 23% | 36% | 4% |
|  | all | 73% | 24% | 37% | 3% |
| *medium* | neurological | 92% | 30% | 61% | 0% |
|  | all | 88% | 35% | 66% | 4% |
| *high* | neurological | 92% | 88% | 79% | 50% |
|  | all | 95% | 71% | 77% | 43% |

Statistics per item:

| item  quality | | **1** | **2** | **3** | **4** | **5** | **6** | **7** | **8** | **9** | **10** | **11** | **12** | **13** | **14** | **15** | **16** | **17** | **18** | **19** | **20** |
| --- | --- | --- | --- | --- | --- | --- | --- | --- | --- | --- | --- | --- | --- | --- | --- | --- | --- | --- | --- | --- | --- |
| *low* | neurological | 100% | 100% | 96% | 50% | 21% | 71% | 38% | 93% | 86% | 43% | 7% | 4% | 79% | 64% | 32% | 100% | 0% | 0% | 15% | 4% |
|  | all | 100% | 100% | 90% | 57% | 24% | 76% | 40% | 95% | 86% | 38% | 5% | 5% | 81% | 57% | 33% | 100% | 5% | 0% | 20% | 3% |
| *medium* | neurological | 100% | 100% | 100% | 87% | 53% | 100% | 86% | 100% | 93% | 86% | 13% | 3% | 100% | 80% | 53% | 100% | 50% | 50% | 36% | 0% |
|  | all | 100% | 100% | 98% | 86% | 45% | 95% | 93% | 100% | 95% | 60% | 25% | 8% | 98% | 86% | 50% | 100% | 60% | 62% | 36% | 4% |
| *high* | neurological | 100% | 100% | 97% | 87% | 58% | 100% | 89% | 100% | 95% | 83% | 32% | 18% | 100% | 79% | 53% | 100% | 61% | 56% | 44% | 11% |
|  | all | 100% | 100% | 100% | 93% | 71% | 100% | 100% | 100% | 100% | 86% | 71% | 57% | 100% | 86% | 50% | 100% | 86% | 71% | 71% | 43% |

# **Supplemental S2 – Table of all included studies**

1. **Neurological studies**

| **Author** | **Subjects** | **Matching of cohorts** | **Walking detection  method** | **Segment detection method** | **Step detection  method** | **Specific condition** | **Follow-up** | **IMU/**  **frequency**  **( Hz)** | **other sensor used for: additional parameters / data analysis** | **Sensor body position** | **Sequence of steps** | **Speed** | **Speed deccorelation attempt?** | **Surface (level, unleveled, obstacle, slope, treadmill)** | **U-turn** | **Sit to stand transition** | **Definition of steps kept for analysis** |
| --- | --- | --- | --- | --- | --- | --- | --- | --- | --- | --- | --- | --- | --- | --- | --- | --- | --- |
| Andrzejewski et al. 2016 | 15 HD patients  vs 5 older healthy participants | no | NS | not needed | NS | no | none; ambulatory | PAMWare, BioSensics, Cambridge, MA / 50 Hz | no / no | sternum | laboratory/ distance: 6m; | self-selected | no | unlevelled (laboratory); ambulatory | yes (laboratory); ambulatory | yes (laboratory); ambulatory | NS (laboratory); ambulatory |
|  |  |  |  |  |  |  |  |  |  |  | ambulatory: 6 days |  |  |  |  |  |  |
| Bregou Bourgeois et al. 2014 | 14 patients with cerebral palsy  vs 15 typically developed children | no | automatic:  Mariani, 2012 | automatic:  for U-turn (Mariani 2010) | automatic:  Mariani, 2012 | no | none | Physilog system, GaitUp, Lausanne, Switzerland / 200 Hz | no / no | both feet | distance: 200m | self-selected | no | unlevelled | yes | no | NS |
| Brodie, Canning, et al. 2015 | 10 PD patients ON medication  vs 10 older healthy participants  vs 10 young healthy participants | age | NS | NS | NS: custom | no | none | Opal, APDM, Inc., Portland, OR, USA / 128 Hz | no / no | lower back | distance: 21m | self-selected | assess correlation of parameters to speed | unlevelled | yes | no | first and last 2m removed |
| Chini et al. 2016 | 16 degenerative spinocerebellar ataxia patients vs 16 healthy participants  Correlation with International Cooperative Ataxia Rating Scale total, balance and posture; risk of falling; and disease duration | age; gender;  speed | automatic:  BTS G-STUDIO® software | not needed | automatic:  BTS G-STUDIO® software | no | none | G-Sensor, BTS Bioengineering S.p.A., Italy / 50 Hz | no / no | lower back | distance: 20m | self-selected  (low speed for controls) | no | unlevelled | yes | no | 8 middle strides |
| Collett et al. 2014 | 16 HD patients with Unified HD Rating Scale-Total Motor Score > 5 and Unified HD Rating Scale- Total Functional Capacity = 7-13 vs 12 HD patients with Unified HD Rating Scale- Total Functional Capacity ≤ 6  vs 7 pre-HD patients with Unified HD Rating Scale-Total Motor Score ≤ 5 vs 22 older healthy participants  correlated with disease burden scores (35 patients)  correlated with Unified HD Rating Scale-Total Motor Score (35 patients) | age | automatic: inverted pendulum model (Zijlstra 2003); Esser 2014 | not needed | automatic: inverted pendulum model (Zijlstra 2003); Esser 2014 | no | none | Pi-Node, Philips, The Netherlands / 100 Hz | no / no | lower back | distance: 10m | self-selected | no | unlevelled | no | no | 6 middle strides kept |
| Del Din, Godfrey, and Rochester 2015 | 30 PD patients (Hoehn and Yahr I-III) vs 30 older healthy participants | age | automatic: McCamley et al, 2012 optimized (Matlab algorithm) | not needed | automatic: McCamley et al, 2012 optimized (Matlab algorithm) | no | none | Axivity AX3, York, UK / 50 Hz | no / no | lower back | distance: 10m | self-selected | no | unlevelled | no | no | first and last 4 steps removed |
| Del Din et al. 2016 | 47 PD patients (Hoehn and Yahr I-III) vs 50 older healthy participants |  | NS (laboratory); automatic (ambulatory): NS (laboratory); Lyons 2005 (ambulatory) | not needed | NS | no | none; ambulatory | Axivity AX3, York, UK / 100 Hz | no / no | lower back | laboratory/distance: 10m; | self-selected | no | unlevelled; ambulatory | no (laboratory); ambulatory | no (laboratory); ambulatory | NS (laboratory);  NA (ambulatory) |
|  |  |  |  |  |  |  |  |  |  |  | ambulatory: 7 days |  |  |  |  |  |  |
|  |  |  |  |  |  |  |  |  |  |  |  |  |  |  |  |  |  |
| Demonceau et al. 2015 | 32 PD patients (Hoehn and Yahr II-III) vs 32 PD patients Hoehn and Yahr < II) vs 32 older healthy participants | no | automatic:  Locometrix software | not needed | automatic: Locometrix software | no | none | Locometrix, Centaure Metrix, Evry, France / 100 Hz | no / no | lower back | distance: 36m | self-selected | no | unlevelled | no | no | 3m |
| Doi et al. 2015 | 109 patients with severe white matter lesions vs 451 patients with non-severe white matter lesions | no | automatic:  inverted pendulum (Zjilstra, 2003) | NS | automatic:  inverted pendulum (Zjilstra, 2003) | dual task | none | MVP-RF8, MicroStone, Nagano, Japon / 200 Hz | no / no | lower back | distance: 15m | self-selected | no | unlevelled | no | no | 2m |
| Ellis et al. 2015 | 12 PD patients vs 12 older healthy participants | no | automatic: machine learning (explained in article) | automatic: machine learning (explained in article) | automatic: machine learning (explained in article) | no | none | smartphone / 100 Hz | yes / no | left foot | distance: 26m | self-selected; average speed; 110% average speed | 3 different speeds: discussion of parameters and ICC correlation | unlevelled | yes | no | NS |
| Gillain et al. 2016 | 15 patients with mild cognitive impairment at risk of AD vs 8 patients with mild cognitive impairment not at risk of AD | no | automatic:  Locometrix software | automatic: Locometrix, Centaure Metrix, Evry, France | automatic: Locometrix software | dual task | laboratory follow-up | Locometrix, Centaure Metrix, Evry, France / 50 Hz | no / no | lower back | distance: 80m | self-selected | no | unlevelled | yes | no | middle 23,48s kept |
| Hatanaka et al. 2016 | 13 PD patients (Hoehn and Yahr I)  vs 31 PD patients (Hoehn and Yahr II) vs 68 PD patients (Hoehn and Yahr III) vs 11 PD patients (Hoehn and Yahr IV) vs 20 Progressive Supranuclear Palsy patients vs 24 older healthy participants Ccorrelated with Hoehn and Yahr stages | age | automatic:  Yoneyama, 2013 and 2014 | not needed | automatic: Yoneyama, 2013; Yoneyama, 2014 | no | none | Mimamori-Gait System, LSI Medience Corp., Tokyo / 100 Hz | no / no | lower back | distance: 10m | self-selected | no | unlevelled | no | no | NS |
| Henderson et al. 2016 | 55 mild idiopathic PD patients receiving rivastigmine vs 59 mild idiopathic PD patients receiving placebo | no (randomization) | NA: heel strike peaks in acceleration signals | not needed | NS: heel strike peaks in acceleration signals | dual task | laboratory follow-up | Dynaport Hybrid, McRoberts, The Hague, The Netherlands / 100 Hz | no / no | lower back | distance: 22m | self-selected | no | unlevelled | no | no | 2m |
| Howell, Osternig, and Chou 2015 | 10 patients with concussion vs 7 healthy participants | no | automatic: inverted pendulum (Zjilstra, 2003) (aAP) | not needed | automatic: inverted pendulum (Zjilstra, 2003) | dual task | laboratory follow-up (up to 2 months after concussion) | Opal, APDM, Inc., Portland, OR, USA / 128 Hz | no / no | lower back | distance: NS | self-selected | speed as a covariate in two-way mixed effects analysis of covariance (ANCOVA) | unlevelled | no | no | NS |
| Hsu et al. 2014 | 21 AD patients vs 50 older healthy participants | no | automatic:  explained in article | not needed | automatic: explained in article | dual task | none | self-made / 100 Hz | no / no | both feet | distance: 40m | self-selected | no | unlevelled | no | no | NS |
| Jaywant et al. 2016 | 13 PD patients with gait intervention (gait observation) vs 10 PD patients without gait intervention (control video observation) | no | automatic: EMG Works® software (heel strike and toe off peaks in acceleration signals) | not needed | automatic: EMG Works® software (heel strike and toe off peaks in acceleration signals) | dual task | laboratory follow-up | Activinsights Ltd / 100 Hz | no / no | one shank | distance: 10m and 20m; 16m; 18.8m and 13.8m | self-selected | no | unlevelled | both | no | NS |
| Kleiner et al. 2015 | 35 PD patients (OFF medication) after adapted multiple point stimulation vs before adapted multiple point stimulation vs 35 older healthy participants | no | automatic:  BTS G- STUDIO® software | NS | automatic:  BTS G- STUDIO® software | no | none | G-Sensor, BTS Bioengineering S.p.A., Italy / 50 Hz | no / no | lower back | distance: NS | self-selected | NS | unlevelled | NS | no | NS |
| Lauretani et al. 2016* | One PD patient with mild cognitive impairment and falls after dopamine intake vs before dopamine intake | NA | NS | NS | NS | no | laboratory follow-up | Free4Act, LorAn-Engineering, Bologna, Italy / NA | no / no | lower back | distance: 10m | self-selected | no | unlevelled | no | no | NS |
| Mancini et al. 2016 | 10 PD patients (Hoehn and Yahr I-III) OFF medication vs 12 older healthy participants | age | automatic: pressure insoles | automatic: pressure insoles | automatic: pressure insoles | no | none | Opal, APDM, Inc., Portland, OR, USA / 128 Hz | yes / no | lower back + both shanks | distance: 1 step | self-selected | no | unlevelled | no | no | first step removed |
| Martinez-Ramirez et al. 2016 | 11 frail older healthy participants with mild cognitive impairment  vs 20 frail older healthy participants without mild cognitive impairment  vs 10 older healthy participants | no | automatic: extensive peak detection and wavelet (explained in article) | not needed | automatic: extensive peak detection and wavelet (explained in article) | no | none | Xsens MTw, Xsens Technologies, Enschede, The Netherlands / 100 Hz | no / no | lower back | distance: 7m | self-selected | no | unlevelled | no | no | 1m removed |
| Martino Cinnera et al. 2015* | One patient with traumatic brain injury after vs before treatment | NA | automatic: optoelectronic device (Optogait®) | automatic: optoelectronic device (Optogait®) | automatic: optoelectronic device (Optogait®) | no | laboratory follow-up | Gyko, Microgate, Italy / 200 Hz | yes / no | lower back | distance: 80m | self-selected | no | unlevelled | yes | no | middle 23,48s kept |
| Matsushima et al. 2015 | 51 patients with spinocerebellar ataxia or multiple system atrophy with predominant spinocerebellar ataxia vs 56 older healthy participants | NS | NS | not needed | NS | no | none | Jukudai Mate; Kissei Comtec Co., Matsumoto, Japan / 20 Hz | no / no | lower back | distance: 10m | self-selected | no | unlevelled | no | no | middle 6,4s kept |
| Mirelman et al. 2014 | 67 patients with mild cognitive impairment vs 280 older healthy participants | no | automatic:  Weiss, 2010 and 2011 | automatic:  Weiss, 2010; Weiss, 2011 | automatic:  Weiss, 2010 and 2011 | no | none | Dynaport Hybrid, McRoberts, The Hague, The Netherlands / 100 Hz | no / no | lower back | distance: 16 feet | self-selected | no | unlevelled | yes | yes | NS |
| Motta et al. 2016 | 80 patients with MS vs 50 healthy participants  24 patients with secondary progressive MS vs 56 patients with relapsing-remitting MS | age; gender | NS | NS | NS | no | none | Xsens MTw, Xsens Technologies, Enschede, The Netherlands / 100 Hz | no / no | lower back + both thighs + both shanks + both feet | time: 1min | self-selected | no | unlevelled | yes | no | NS |
| Pan et al. 2015 | 11 PD patients (Unified PD Rating Scale-Gait >2)  vs 18 PD patients (Unified PD Rating Scale -Gait <2) | no | automatic:  Dr PD application | automatic:  Dr PD application | automatic:  Dr PD application | no | none | smartphone / 100 Hz | no / no | one ankle | distance: 25 feet | self-selected | no | unlevelled | 360° | no | first and last steps removed |
| Perrochon et al. 2015 | 16 dementia patients after multicomponent exercise vs 16 dementia patients before multicomponent exercise | no | automatic: Locometrix®  software | not needed | automatic: Locometrix® software | no | laboratory follow-up | Locometrix, Centaure Metrix, Evry, France / 100 Hz | no / no | lower back | distance: 20m | self-selected | no | unlevelled | no | no | NS |
| Reynard et al. 2014 | 83 patients with moderate paresis of lower extremities vs 40 older healthy participants | no | not needed | not needed | not needed | no | none | Physilog system, GaitUp, Lausanne, Switzerland / 200 Hz | no / no | lower back | time: 30s | self-selected | assess correlation of parameters to cadence | unlevelled | no | no | 5s |
| Saether et al. 2014 | 41 patients with cerebral palsy vs 29 typically developed children correlated with disease and severity of cerebral palsy (Gross Motor Function Classification System) (41 patients) | age | automatic: photoelectric cells | automatic: not needed | automatic: photoelectric cells | no | none | Xsens MTw, Xsens Technologies, Enschede, The Netherlands / 100 Hz | no / no | lower back | distance: 5m | self-selected | 3 different speeds: linear mixed model with speed and group as fixed effects (participant as a random effect) | unlevelled | no | no | first and last 5m removed |
| Saether et al. 2015 | Correlation with control sitting in spastic cerebral palsy (26 patients) | NA | NS | not needed | NS | NS | NS | NS | NS / NS | NS | NS | NS | NS | unlevelled | no | no | NS |
| Schmitz-Hubsch et al. 2016 | 8 patients with mild to moderate spinocerebellar ataxia vs 9 young healthy participants | age; height | automatic:  Mobility Lab® | not needed | automatic:  Mobility Lab® | no | none | Opal, APDM, Inc., Portland, OR, USA / 128 Hz | yes / no | one shank | distance: 8,1m | very slow; slow; fast; fastest; self-selected | 5 different speeds: discussion of parameters and between method consistency | unlevelled | no | no | first and last 1.5m removed |
| Sejdic et al. 2014 | 10 PD patients vs 11 peripheral neuropathy patients vs 14 older healthy participants | no | automatic: 3-D optical motion capture system | automatic: 3-D optical motion capture system | automatic: 3-D optical motion capture system | no | none | MMA7260Q, Freescale Semiconductor / 100 Hz | yes / no | lower back | time: 3min | self-selected | no | treadmill | no | no | NS |
| Shirai et al. 2015 | 25 patients with spinocerebellar ataxia vs 25 PD patients  vs 25 older healthy participants | no | NS | NS | NS | no | none | Mimamori-Gait System, LSI Medience Corp., Tokyo / 100 Hz | no / no | upper back + lower back | time: 6min | self-selected | no | unlevelled | yes | no | NS |
| Summa et al. 2016 | 20 patients with cerebral palsy  vs 20 typically developed children  correlated with disease and severity of cerebral palsy (Gross Motor Function Classification System) (20 patients) | age; gender | automatic:  Masci, 2013 | not needed | automatic:  Masci, 2013 | no | none | Opal, APDM, Inc., Portland, OR, USA / 128 Hz | no / no | head + sternum + lower back | distance: 10m | self-selected | no | unlevelled | no | no | NS |
| Weiss et al. 2014 | 40 faller PD patients (Hoehn and Yahr I-IV) vs 67 non faller PD patients (Hoehn and Yahr I-IV) | no | automatic:  Weiss, 2011 | automatic:  Weiss, 2011 | automatic:  Weiss 2011 | no | ambulatory | Dynaport Hybrid, McRoberts, The Hague, The Netherlands / 100 Hz | no / no | lower back | ambulatory:  3 days | self-selected | no | ambulatory | ambulatory | ambulatory | NA (ambulatory) |
| Weiss et al. 2015a | 107 PD patients (Hoehn and Yahr I-IV) separated into 2 groups of low and high score for: cognitive, attention and executive function. | no | automatic:  Weiss 2011 | not needed | automatic:  Weiss, 2011 | no | ambulatory | Dynaport Hybrid, McRoberts, The Hague, The Netherlands / 100 Hz | no / no | lower back | ambulatory:  3 days | self-selected | no | ambulatory | ambulatory | ambulatory | NA (ambulatory) |
| Weiss et al. 2015b | 28 PD patients with freezing of gait  vs 44 PD patients without freezing of gait correlated with the new freezing of gait questionnaire (72 PD patients) | no | automatic:  Weiss, 2011, 2013 and 2014 | automatic:  Weiss, 2011, 2013, 2014 | automatic:  Weiss, 2011, 2013 and 2014 | no | ambulatory | Dynaport Hybrid, McRoberts, The Hague, The Netherlands / 100 Hz | no / no | lower back | ambulatory:  3 days | self-selected | no | ambulatory | ambulatory | ambulatory | NA (ambulatory) |
| Yoneyama et al. 2015 | 13 patients with severe mild cognitive impairment and dementia  vs 13 patients with non-severe mild cognitive impairment and dementia  vs 13 patients with severe PD  vs 13 patients with non-severe PD  vs 13 older healthy participants | age | automatic:  Yoneyama 2014 | not needed | automatic: Yoneyama, 2014 | no | ambulatory | Mimamori-gait system, LSI Medience Corp. / 100 Hz | no / no | lower back | ambulatory:  1 day | self-selected | no | ambulatory | ambulatory | ambulatory | NA (ambulatory) |
| Zollinger et al. 2016 | 10 patients with unilateral spastic cerebral palsy  vs 10 typically developed children | age; height; BMI | automatic: heel strike and toe off determined from antero-posterior and cranio-caudal acceleration signals (Jasiewicz, 2006) | not needed | automatic: heel strike and toe off determined from antero-posterior and cranio-caudal acceleration signals (Jasiewicz, 2006) | no | none | Xsens MTw, Xsens Technologies, Enschede, The Netherlands / 100 Hz | no / no | lower back + one shank | unlevelled/ distance: 30m; | self-selected (unlevelled); 0.97 ie average for CP in the litterature (treadmill) | 2 different speeds: subgroup analysis | unlevelled; treadmill | no | no | NS |
|  |  |  |  |  |  |  |  |  |  |  | treadmill: at least 10 strides |  |  |  |  |  |  |

Abbreviations: HD: Huntington Disease; PD: Parkinson disease; AD: Alzheimer disease; MS: multiple sclerosis; NS: not specified; NA: not applicable; ICC: intraclass correlation coefficient

1. **Age and morphology-related studies**

| **author** | **Patients** | **matching** | **walking bout detection method** | **segment detection method** | **Step detection method** | **Specific condition** | **Follow-up** | **IMU - frequency (Hz)** | **other sensor used for: additional parameters / data analysis** | **Sensor body position** | **Sequence of steps** | **speed** | **speed deccorelation attempt?** | **Surface (level, unleveled, obstacle, slope, treadmill)** | **U-turn?** | **Sit to stand transition?** | **definition of steps kept for analysis:** |
| --- | --- | --- | --- | --- | --- | --- | --- | --- | --- | --- | --- | --- | --- | --- | --- | --- | --- |
| Arvin et al. 2016 | 18 elderly healthy subjects  *vs* 18 young healthy subjects | no | automatic: Pressure sensors | not needed | automatic: Pressure sensors | predetermined preferred step width; 50% narrower step width | no follow-up | Dynaport Hybrid, McRoberts, The Hague, The Netherlands - 100Hz | no / yes | lower back | time: 2,5min | self-selected | no | treadmill | no | no | NS |
| Bragge et al. 2014 | 16 patients after  *vs* before weight loss following bariatric surgery | no | automatic: NS | NS | automatic: Photoelectric cells | no | laboratory follow-up (before and after bariatric surgery) | ME6000 T16, Mega Electronics Ltd; Kuopio, Finland - 1000Hz | no / yes | one shank | distance: 10m | 1,2m/s and 1,5m/s | 2 different speeds: subgroup analysis | unlevelled | no | no | first and last 3,4m removed |
| Brodie et al. 2015a | 7 faller elderly healthy subjects  *vs* 11 non-faller elderly healthy subjects | age; height; Body Mass Index | wavelet-based decision tree algorithm designed for remote monitoring: aCC peaks in the level 4 and 5 Daubechies fifth- order wavelet decomposition | wavelet-based decision tree algorithm designed for remote monitoring: aCC peaks in the level 4 and 5 Daubechies fifth- order wavelet decomposition | wavelet-based decision tree algorithm designed for remote monitoring: aCC peaks in the level 4 and 5 Daubechies fifth- order wavelet decomposition | no | ambulatory | Senior Mobility Monitor, Philips Research Europe, Netherlands - 50Hz | no / no | sternum | ambulatory: 8 weeks | self-selected | no | ambulatory | ambulatory | ambulatory | NA (ambulatory) |
| Brodie et al. 2015b | 35 faller elderly healthy subjects  *vs* 61 non faller elderly healthy subjects  *vs* 96 elderly healthy subjects  *vs* 29 young healthy subjects | no | NS | not needed | NS | no | no follow-up | Opal, APDM, Inc., Portland, OR, USA - 128Hz | no / no | lower back | distance: 20m | self-selected | subgroup analysis: one above median, one below median) | unlevelled | no | no | first and last 2,5m removed |
| Buckinx et al. 2015 | 36 elderly healthy subjects at time 0  *vs* a 2-year follow-up | no | not needed | not needed | NS | no | laboratory follow-up | Locometrix, Centaure Metrix, Evry, France - NAHz | no / no | lower back | distance: 20m | self-selected | no | unlevelled | no | no | NS |
| Cui et al. 2014 | 39 faller elderly healthy subjects  *vs* 42 non-faller elderly healthy subjects |  | not needed | NS | not needed | with harness; obstacle negotiation walk | no follow-up | Dynaport Hybrid, McRoberts, The Hague, The Netherlands - 100Hz | no / no | lower back | time: 1min | self-selected | no | unlevelled | NA | NA | NS |
| Galan-Mercant and Cuesta-Vargas 2014 | 14 frail elderly healthy subjects  *vs* 16 non frail elderly healthy subjects | no | not needed | automatic: Salarian, 2009 | not needed | no | no follow-up | smartphone (Iphone 4) - 32Hz | no / no | lower back | distance: 10m | fastest | no | ambulatory | yes | yes | NS |
| Greene et al. 2014 | 215 frail elderly healthy subjects  *vs* 184 non frail elderly healthy subjects | no | automatic: Greene 2010; Greene 2012 | not needed | automatic: Greene, 2010; | no | no follow-up | Shimmer, Dublin, Ireland - 102,4Hz | no / yes | both shanks | distance: 6m | fastest | no | unlevelled | yes | yes | NS |
| Howcroft et al. 2014 | 11 elderly healthy subjects during double tasking  *vs* single tasking | NA | not needed | not needed | not needed | dual task | no follow-up | X16-1C, Gulf Coast Data Concepts, Waveland, MS - 50Hz | no / no | lower back | distance: 25 feet | self-selected | no | unlevelled | no | no | NS |
| Howcroft et al. 2016 | 24 faller elderly healthy subjects  *vs* 76 non-faller elderly healthy subjects | NA | automatic: Pressure sensors | not needed | automatic: Pressure sensors | dual task | no follow-up | X16-1C, Gulf Coast Data Concepts, Waveland, MS - 50Hz | no / yes | head + lower back + both ankles | distance: 25 feet | self-selected | assess correlation of parameters to speed (one above 0,7) | unlevelled | no | no | first and last 1m are removed |
| Ihlen, Weiss, Beck, et al. 2016 | 31 faller elderly healthy subjects  *vs* 39 non-faller elderly healthy subjects | age | automatic: signal magnitude area (SMA) threshold based filter combined with a frequency based filter (Weiss et al., 2013, 2011) | automatic: SMA threshold based filter combined with a frequency based filter (Weiss et al., 2013, 2011) | automatic: signal magnitude area (SMA) threshold based filter combined with a frequency based filter (Weiss et al., 2013, 2011) | no | ambulatory | Dynaport Hybrid, McRoberts, The Hague, The Netherlands - 100Hz | no / no | lower back | ambulatory: 3 days | self-selected | no | ambulatory | ambulatory | ambulatory | NA (ambulatory) |
| Ihlen, Weiss, Bourke, et al. 2016 | 32 faller elderly healthy subjects  *vs* 39 non faller elderly healthy subjects | no | not needed | automatic: threshold-based algorithm (Weiss, 2011) | not needed | no | ambulatory | Dynaport Hybrid, McRoberts, The Hague, The Netherlands - 100Hz | no / no | lower back | ambulatory: 3 days | self-selected | no | ambulatory | ambulatory | ambulatory | NA (ambulatory) |
| Kobsar et al. 2014 | 41 elderly healthy subjects  *vs* 41young healthy subjects | no | automatic: local minima method on antero-posterior acceleration signal | not needed | automatic: local minima method on antero-posterior acceleration signal | no | no follow-up | self-made - 100Hz | no / no | lower back | time: 10min | self-selected | no | unlevelled | yes | no | 30s |
| Martinez-Ramirez et al. 2015 | 65 frail elderly healthy subjects  *vs* 327 pre frail elderly healthy subjects  *vs* 326 elderly healthy subjects | no | automatic: extensive peak detection and wavelet (explained in article) | not needed | automatic: extensive peak detection and wavelet (explained in article) | no | no follow-up | Xsens MTw, Xsens Technologies, Enschede, The Netherlands - 100Hz | no / no | lower back | distance: 5m | self-selected | no | unlevelled | no | no | 1m |
| Martinikorena et al. 2016 | dorrelation to strength | NA | NS | not needed | NS | no | no follow-up | Xsens MTw, Xsens Technologies, Enschede, The Netherlands - 100Hz | no / no | lower back | distance: 7m | self-selected | no | unlevelled | no | no | first and last 1m removed |
| Matsumoto et al. 2016 | 41 elderly healthy subjects with locomotive syndrome  *vs* 182 elderly healthy subjects without locomotive syndrome | no | automatic: Opto Gait | automatic: Opto Gait | automatic: Opto Gait | no | laboratory follow-up | MVP-RF8, MicroStone, Nagano, Japon - 200Hz | yes (OptoGait) / NA | lower back | distance: 9m | self-selected | no | unlevelled | no | no | first and last 2m removed |
| Pau et al. 2014 | 17 elderly healthy subjects pre and post light physical activity  *vs* 17 elderly healthy subjects pre and post vigorous physical activity | NA | automatic: BTS G-STUDIO software | not needed | automatic: BTS G-STUDIO software | eyes closed | laboratory follow-up | G-Sensor, BTS Bioengineering S.p.A., Italy - 50Hz | no / no | lower back | distance: 8m | self-selected | no | unlevelled | no | no | NS |
| Reginatto et al. 2015* | One faller elderly healthy subject (poly-pathological)  *vs* non faller elderly healthy subject (NA)  One faller EHS (CV pathology)  *vs* non faller EHS (NA) | age | automatic: Fraccaro, 2014 | not needed | automatic: Fraccaro, 2014 | no | ambulatory | Shimmer, Dublin, Ireland - 102,4Hz | no / no | both ankles | ambulatory: 6 days | self-selected | no | ambulatory | ambulatory | ambulatory | NA (ambulatory) |
| Rivolta et al. 2015 | correlation to falling risk (13 patients) | no | manual: NS | not needed | manual: NS | no | no follow-up | GENEActiv, Activinsights Limited, United Kingdom - 50Hz | no / no | sternum | distance: 10m | self-selected | no | unlevelled | no | no | NS |
| Scaglioni-Solano and Aragon-Vargas 2015 | 35 elderly healthy subjects  *vs* 22 young healthy subjects | no | automatic: inverted pendulum (Zjilstra, 2003) (antero-posterior acceleration signal) | not needed | automatic: inverted pendulum (Zjilstra, 2003) (antero-posterior acceleration signal) | no | no follow-up | Technaid SL - 50Hz | no / no | head + lower back | distance: 15m | self-selected | no | 3 level types: unlevelled; 8% slope; 20% slope | no | no | first and last 2,5m are removed |
| Sheehan et al. 2014 | correlation to balance decline | NA | automatic: Greene, 2010; Greene, 2012 | not needed | automatic: Greene, 2010; Greene, 2012 | no | laboratory follow-up | Shimmer, Dublin, Ireland - 102,4Hz | no / no | both shanks | distance: 6m | fastest | no | unlevelled | yes | yes | NS |
| Shin, An, and Yoo 2015 | patients with poor binocular visual acuity (number not specified)  vs patients with good binocular visual acuity (number not specified) | NA | not needed | not needed | not needed | no | no follow-up | Fit Dot Life, Suwon, Korea - 32Hz | no / no | lower back | distance: 11,5m | self-selected | no | unlevelled | yes | no | 2m |
| Toebes et al. 2015 | elderly healthy subjects with fear of falling (number not specified)  *vs* elderly healthy subjects without fear of falling (number not specified);  faller elderly healthy subjects (number not specified)  *vs* non faller elderly healthy subjects (number not specified) | no | automatic: inverted pendulum (Zjilstra, 2003) (antero-posterior acceleration signal) | not needed | automatic: inverted pendulum (Zjilstra, 2003) (antero-posterior acceleration signal) | no | no follow-up | Dynaport Hybrid, McRoberts, The Hague, The Netherlands - 100Hz | no / no | upper back | time: 12-17min | 1,1m/s | no | treadmill | no | no | 150 last steps kept |
| Valenti, Bonomi, and Westerterp 2015 | correlation to age (35 elderly healthy subjects) | NA | NS: aCC peak | not needed | NS: aCC peak | no | no follow-up | GT3X++ (ActiGraph, Pensacola, FL) - 60Hz | no / no | lower back | time: 2min | optimal speed (minimizing energy consumption) | 6 different speeds: discussion of parameters | treadmill | no | no | first 3min are removed |
| van Schooten et al. 2016 | correlation to time to first fall | NA | automatic: Dijkstra, 2010 | not needed | automatic: Rispens, 2015; Van Schooten, 2015 | no | ambulatory | Dynaport MoveMonitor, McRoberts, The Hague, The Netherlands - 100Hz | no / no | lower back | ambulatory: 8 days | self-selected | no | ambulatory | ambulatory | ambulatory | NA (ambulatory) |
| Zakaria et al. 2015 | 21 patients with high fall risk  vs 17 patients with low fall-risk | no | manual: explained in the article | manual: explained in the article | not needed | no | no follow-up | self-made (Acc: MMA7260Q Freescale semiconductor inc. Texas, USA; gyr: Murata, Kyoto, Japan, ENC-03R and XV-3500CB, Epson Toyocom, Miyazaki Epson Corp.) - 100Hz | no / no | lower back | distance: 6m | fastest | no | unlevelled | yes | yes | 0 |

1. **Rheumatology studies**

| **author** | **Patients** | **matching** | **walking bout detection method** | **segment detection method** | **Step detection method** | **Specific condition** | **Follow-up** | **IMU - frequency (Hz)** | **other sensor used for: additional parameters / data analysis** | | **Sensor body position** | | **Sequence of steps** | | **speed** | | **speed deccorelation attempt?** | | **Surface (level, unleveled, obstacle, slope, treadmill)** | | **U-turn?** | | **Sit to stand transition?** | | **definition of steps kept for analysis:** | |
| --- | --- | --- | --- | --- | --- | --- | --- | --- | --- | --- | --- | --- | --- | --- | --- | --- | --- | --- | --- | --- | --- | --- | --- | --- | --- | --- |
| Arvin et al. 2016 | 17 elderly healthy subjects after hip abductor fatigue  *vs* 17 elderly healthy subjects before hip abductor fatigue | NA | automatic: peak of antero-posterior acceleration signal (zijlstra’s inverted pendulum model) | not needed | automatic: peak of antero-posterior acceleration signal (zijlstra’s inverted pendulum model) | no | no follow-up | Dynaport Hybrid, McRoberts, The Hague, The Netherlands - 100Hz | no / no | | lower back | | time: 5min | | self-selected | | no | | treadmill | | no | | no | | NS | |
| Barrois et al. 2015 | osteoarthritis with low WOMAC score (WOMAC 65-96)  *vs* osteoarthritis with medium WOMAC score (WOMAC 33-64)  *vs* osteoarthritis with severe WOMAC score (WOMAC 1-32)  *vs* 12 elderly healthy subjects |  | manual: gCC peaks and zeros | not needed | manual: gCC peaks and zeros | no | no follow-up | Xsens MTw, Xsens Technologies, Enschede, The Netherlands - 100Hz | no / no | lower back | | distance: 20m | | self-selected | | no | | unlevelled | | yes | | no | | NS | |  |
| Bolink et al. 2015a | 20 patients with advanced unilateral hip osteoarthritis  *vs* 20 patients with advanced unilateral knee osteoarthritis  *vs* 20 healthy subjects |  | automatic: Gonzalez, R.C., et al., 2010. | not needed | automatic: Greene, 2010; | no | no follow-up | MicroStrain, Inertia-Link - 100Hz | no / no | lower back | | distance: 20m | | self-selected | | normalization from interpolation | | unlevelled | | no | | no | | NS | |  |
| Bolink et al. 2015b | 20 patients post-total knee arthroplasty  *vs* pre- total knee arthroplasty | NA | automatic: zero crossing method (Gonzalez, 2006) | not needed | automatic: zero crossing method (Gonzalez, 2006) | no | laboratory follow-up | Microstrain, Inertia-Link - 100Hz | no / no | | lower back | | distance: 20m | | self-selected | | no | | unlevelled | | no | | no | | NS | |
| Bolink et al. 2016 | 18 low function group pre *vs* post total hip arthroplasty  *vs* 18 high function group pre *vs* post total hip arthroplasty  correlation to WOMAC score | no | automatic: Gonzalez, 2010 | not needed | automatic: Gonzalez, 2010 | no | laboratory follow-up (pre THA, 3 months post THA, 12 months post THA) | MicroStrain, Inertia-Link - 100Hz | no / no | | lower back | | distance: 20m | | self-selected | | no | | unlevelled | | no | | no | | NS | |
| Chopra et al. 2014 | 12 patients with ankle arthroplasty: pathological side *vs* healthy side  *vs*  12 patients with total ankle reconstruction: pathological side *vs* healthy side | no | automatic: Mariani, 2013; Rouhani, 2012: plantar pressure insoles | not needed | automatic: Mariani, 2013; Rouhani, 2012: plantar pressure insoles | no | laboratory follow-up | Physilog system, GaitUp, Lausanne, Switzerland - 200Hz | no / yes | | both feet | | distance: 50m | | self-selected | | no | | unlevelled | | no | | no | | first and last 3 strides removed | |
| Elbaz et al. 2016 | 24 ankle fracture  *vs* 14 healthy subjects | age; BMI | automatic: Poseidon software | not needed | automatic: Poseidon software | no | no follow-up | GaitSmart, ETB, UK - 102,4Hz | no / no | | both thighs + both shanks | | distance: at least 7 strides | | self-selected | | no | | unlevelled | | no | | no | | NS | |
| Henchoz et al. 2015 | 13 chronic low back pain patients  *vs* 13 healthy subjects | age; height; BMI | automatic: heel strike peaks in medio-lateral angular velocity signal (Jasiewicz, 2006) | NS | automatic: heel strike peaks in medio-lateral angular velocity signal (Jasiewicz, 2006) | no | no follow-up | Xsens MTw, Xsens Technologies, Enschede, The Netherlands - 100Hz | no / no | | lower back + one shank | | time: 5min | | self-selected | | 5 different speeds: subgroup analysis (correlation using ANOVAs) | | treadmill | | no | | no | | NA | |
| Hjorth et al. 2014 | 28 large-head metal-on-metal total hip arthroplasty vs 20 elderly healthy subjects | no | automatic: zero crossing method (Gonzalez, 2010); inverted pendulum (Zjilstra, 2003) | not needed | automatic: zero crossing method (Gonzalez, 2010); inverted pendulum (Zjilstra, 2003) | no | no follow-up | MicroStrain, Inertia-Link - 100Hz | no / no | | lower back | | distance: 40m | | self-selected | | no | | unlevelled | | no | | no | | NS | |
| Patterson et al. 2014 | 17 anterior-cruciate ligament reconstruction athlete female  *vs* 17 young healthy subjects | no | automatic: force plates (Greene, 2010) | not needed | automatic: force plates (Greene, 2010) | no | no follow-up | Xsens MTw, Xsens Technologies, Enschede, The Netherlands - 100Hz | no / yes | | one shank | | distance: 15m | | self-selected | | no | | unlevelled | | no | | no | | middle 10 strides kept | |
| Rahman et al. 2015 | 28 patients with total knee arthroplasty pre-op and post-op  *vs* 29 healthy subjects |  | NS | not needed | NS | no | laboratory follow-up (pre-op, 8 weeks post-op, 52 weeks post-op) | GaitSmart, ETB, UK - 102,4Hz | no / no | | both thighs + both shanks | | distance: 20m | | self-selected | | no | | unlevelled | | yes | | no | | one typical stride kept | |
| Rapp et al. 2015 | 15 male post-total hip arthroplasty  *vs* 16 male elderly healthy subjects  *vs* 14 female post- total hip arthroplasty  *vs* 14 female elderly healthy subjects | age; weight; height; BMI | automatic: photo cells (Alge Timing, Lustenau, Austria) | not needed | automatic: photo cells (Alge Timing, Lustenau, Austria) | no | laboratory follow-up (15 days post-THA, 21 days post-THA, 27 days post-THA) | Humotion, Münster, Germany - 100Hz | no / yes | | lower back | | distance: 20,3m | | self-selected; fast | | 2 different speeds and linear mixed model with speed as the dependant variable | | unlevelled | | no | | no | | first and last 4 steps removed | |
| Staab et al. 2014 | 12 knee osteoarthritis subjects  *vs* 7 young healthy subjects | no | automatic: six-camera optoelectronic system | not needed | automatic: six-camera optoelectronic system | no | no follow-up | ADXRS 300, Analog Devices - 1000Hz | no / no | | lower back | | distance: 500m | | self-selected | | 3 different speeds: normalization from interpolation | | treadmill | | no | | no | | NS | |
| Tadano et al. 2016 | 8 severe knee osteoarthritis  *vs* 8 mild knee osteoarthritis  *vs* 10 young healthy subjects | no | automatic: H-gait | not needed | automatic: H-gait | no | no follow-up | H-Gait system - 100Hz | no / no | | lower back + both thighs + both shanks + both feet | | distance: 7m | | self-selected | | no | | unlevelled | | no | | no | | NS | |

Abbreviations: BMI: body mass index; WOMAC: Western Ontario and McMaster Universities Osteoarthritis Index)

# **Supplemental S3 –** Table of discrimination power for the 57 variables included, classified by a semeiological viewpoint.

|  | | CONTROLS | | | | PATHOLOGY | | | | | | | | | | | |
| --- | --- | --- | --- | --- | --- | --- | --- | --- | --- | --- | --- | --- | --- | --- | --- | --- | --- |
|  |  | **Elderly healthy subject** | **Young healthy subject** | **Healthy subject** | **Typically developped child** | **faller and/or frail EHS** | | **Parkinson Disease** | | **MCI, dementia** | | **Spinocerebellar Ataxia** | | **Cerebral Palsy** | | **Osteoarthritis** | |
|  |  | *N* | *N* | *N* | *N* | *dP* | *N* | *dP* | *N* | *dP* | *N* | *dP* | *N* | *dP* | *N* | *dP* | *N* |
| **Speed** | speed (m/s) | 23 | 3 | 1 | 1 | 0,5 | 10 | 0,38 | 21 | 0,71 | 7 | 0,5 | 2 | 0,25 | 4 | 0,4 | 5 |
| **Springiness** | step time (s) | 14 | 4 | 4 | 1 | 0,2 | 10 | 0,23 | 39 | 0,5 | 2 | 0,25 | 4 | 0,25 | 4 | 0,14 | 7 |
|  | foot flat time |  |  | 1 | 1 |  |  |  |  |  |  |  |  | 1 | 1 |  |  |
|  | heel strike time |  |  |  | 1 |  |  |  |  |  |  |  |  | 1 | 1 |  |  |
|  | swing time | 2 | 2 |  | 1 | 0 | 1 | 0 | 8 |  |  | 0 | 1 | 1 | 1 | 1 | 2 |
|  | turn time | 2 |  |  |  | 0,5 | 2 |  |  | 1 | 1 |  |  |  |  |  |  |
|  | step number in turn |  |  |  |  |  |  | 0,28 | 7 |  |  |  |  |  |  |  |  |
| **Sturdiness** | step length | 19 | 2 | 3 | 2 | 0,75 | 4 | 0,43 | 21 | 0,33 | 3 | 0, 67 | 3 | 0,5 | 4 | 0,5 | 4 |
|  | RMS aAP {LB} | 1 |  |  | 1 | 1 | 1 |  |  |  |  |  |  | 1 | 1 |  |  |
|  | RMS aCC {LB} | 2 |  |  | 1 | 1 | 1 |  |  |  |  |  |  | 1 | 1 |  |  |
|  | max aCC {LB} | 1 |  |  |  | 0 | 2 |  |  |  |  |  |  |  |  |  |  |
|  | max aAP {S} |  |  |  |  | 0 | 2 |  |  |  |  |  |  |  |  |  |  |
|  | max aCC {S} |  |  |  |  | 0 | 2 |  |  |  |  |  |  |  |  |  |  |
|  | max aML {S} | 1 |  |  |  | 0 | 2 | 1 | 1 |  |  |  |  |  |  |  |  |
| **Smoothness** | mediolateral angle at heel strike {F} |  |  |  | 1 |  |  |  |  |  |  |  |  | 1 | 1 |  |  |
|  | mediolateral angle at toe off {F} |  |  | 1 | 1 |  |  |  |  |  |  |  |  | 1 | 1 |  |  |
|  | min aAP {LB} | 1 |  |  |  | 0,33 | 3 |  |  |  |  |  |  |  |  |  |  |
|  | min aCC {LB} | 1 |  |  |  | 0,33 | 3 |  |  |  |  |  |  |  |  |  |  |
| **Steadiness** | step autocorrelation coefficient aAP {LB} | 9 | 1 |  |  | 0 | 1 |  |  | 1 | 1 |  |  |  |  |  |  |
|  | step autocorrelation coefficient aCC {LB} | 4 |  |  |  | 0,5 | 4 |  |  | 0,5 | 2 |  |  |  |  |  |  |
|  | step autocorrelation coefficient aML {LB} |  |  |  |  | 0 | 1 |  |  |  |  |  |  |  |  |  |  |
|  | sum of step and stride autocorrelation coefficients {LB} | 3 |  |  |  | 0 | 2 | 0,75 | 4 | 0 | 3 |  |  |  |  |  |  |
|  | stride autocorrelation coefficient aAP {LB} | 2 |  |  |  | 0 | 1 | 0,2 | 10 |  |  |  |  |  |  |  |  |
|  | stride autocorrelation coefficient aCC {LB} | 3 |  |  |  | 0,75 | 4 | 0,6 | 10 | 1 | 1 |  |  |  |  |  |  |
|  | stride autocorrelation coefficient aML {LB} | 1 |  |  |  | 0 | 1 | 0,2 | 10 |  |  |  |  |  |  |  |  |
|  | CV aAP {LB} |  |  |  |  | 0 | 1 |  |  |  |  |  |  |  |  |  |  |
|  | CV aCC {LB} |  |  |  |  | 0 | 1 |  |  |  |  |  |  |  |  |  |  |
|  | CV aML {LB} |  |  |  |  | 0 | 1 |  |  |  |  |  |  |  |  |  |  |
|  | CV double stance time |  |  |  |  | 0 | 1 |  |  |  |  |  |  |  |  |  |  |
|  | CV step length | 1 |  |  | 1 | 0 | 1 | 0,17 | 6 |  |  | 0 | 1 | 1 | 1 |  |  |
|  | CV step time | 13 | 2 | 1 |  | 0,13 | 7 | 0,15 | 13 | 0,5 | 2 | 0 | 1 | 0 | 1 | 0,25 | 4 |
|  | CV swing time | 1 |  |  | 1 | 0 | 1 | 0 | 7 | 1 | 1 |  |  | 1 | 1 |  |  |
| **Stability** | Approximate entropy aCC {LB} | 2 |  |  |  | 0,5 | 4 |  |  | 0 | 1 |  |  |  |  |  |  |
|  | Lyapunov Exponent aAP {LB} | 1 |  |  |  | 0 | 1 | 0 | 2 |  |  |  |  |  |  |  |  |
|  | Lyapunov Exponent aCC {LB} | 1 |  |  |  | 0 | 3 | 0 | 2 |  |  |  |  |  |  |  |  |
|  | Lyapunov Exponent aML {LB} | 1 |  | 1 |  | 0 | 1 | 0 | 2 |  |  | 1 | 1 |  |  |  |  |
|  | power of dominant frequency aAP {LB} |  |  |  |  |  |  | 0 | 10 |  |  |  |  |  |  |  |  |
|  | power of dominant frequency aCC {LB} |  |  |  |  |  |  | 0,8 | 10 |  |  |  |  |  |  |  |  |
|  | power of dominant frequency aML {LB} |  |  |  |  |  |  | 0 | 10 |  |  |  |  |  |  |  |  |
|  | RMS head and lower back ratio aAP {LB + H} |  |  |  |  |  |  |  |  |  |  |  |  | 0 | 1 |  |  |
|  | RMS head and lower back ratio aCC {LB + H} |  |  |  |  |  |  |  |  |  |  |  |  | 0 | 1 |  |  |
|  | RMS head and lower back ratio aML {LB + H} |  |  |  |  |  |  |  |  |  |  |  |  | 0 | 1 |  |  |
|  | RMS aML {LB} | 2 |  |  | 1 | 1 | 2 |  |  |  |  |  |  | 1 | 1 |  |  |
|  | ROM lML {LB} | 2 | 2 | 1 | 1 |  |  | 0 | 5 |  |  |  |  | 1 | 1 |  |  |
|  | ROM anAP {LB} |  |  | 1 |  |  |  |  |  |  |  |  |  |  |  | 0,5 | 4 |
|  | min aML {LB} | 1 |  |  |  | 0,33 | 3 |  |  |  |  |  |  |  |  |  |  |
|  | max aML {LB} | 1 |  |  |  | 0,33 | 3 |  |  |  |  |  |  |  |  |  |  |
| **Symmetry** | sym. autocorr. P1/P2 {LB} | 2 |  |  |  | 0 | 2 | 1 | 2 | 0,5 | 4 |  |  |  |  |  |  |
|  | autocorrelation coefficient right/left symmetry aAP {LB} |  |  |  | 1 | 1 | 1 |  |  |  |  |  |  | 1 | 1 |  |  |
|  | autocorrelation coefficient right/left symmetry aCC {LB} |  |  |  | 1 | 1 | 1 |  |  |  |  |  |  | 1 | 1 |  |  |
|  | autocorrelation coefficient right/left symmetry aML {LB} |  |  |  | 1 | 1 | 1 |  |  |  |  |  |  | 1 | 1 |  |  |
|  | harmonic ratio aAP {LB} | 2 |  |  |  | 0 | 3 | 0,58 | 12 |  |  |  |  |  |  |  |  |
|  | harmonic ratio aCC {LB} | 2 |  |  |  | 0,16 | 6 | 0,5 | 12 | 0 | 1 |  |  |  |  |  |  |
|  | harmonic ratio aML {LB} | 4 | 1 |  |  | 0,33 | 3 | 0,33 | 12 |  |  |  |  |  |  |  |  |
|  | step time right/left symmetry |  |  |  |  |  |  | 0 | 3 |  |  |  |  |  |  | 0 | 3 |
|  | swing time right/left symmetry |  |  |  |  |  |  | 0,29 | 7 |  |  |  |  |  |  |  |  |
| **Synchronisation** | double stance time | 3 |  |  | 1 | 0 | 1 | 0,2 | 5 |  |  | 0 | 2 | 0,5 | 2 |  |  |

Abbreviations: aAP: antero-posterior acceleration; aML: medio-lateral acceleration; aCC: cranio-caudal acceleration; lML: length in the mediolateral direction; anAP: angle around the antero-posterior axis; CV: coefficient of variation; ROM: range of motion; RMS: root mean square; dP: discrimination power (percentage of analysis in which the parameter was significant calculated to the number of times the parameter was assessed); N: number of studies in which the parameter was assessed.

*References*

Andrzejewski, Kelly L., Ariel V. Dowling, David Stamler, Timothy J. Felong, Denzil A. Harris, Cynthia Wong, Hang Cai, et al. 2016. “Wearable Sensors in Huntington Disease: A Pilot Study.” *Journal of Huntington’s Disease* 5 (2): 199–206. doi:10.3233/JHD-160197.

Arvin, Mina, Masood Mazaheri, Marco J. M. Hoozemans, Mirjam Pijnappels, Bart J. Burger, Sabine M. P. Verschueren, and Jaap H. van Dieen. 2016. “Effects of Narrow Base Gait on Mediolateral Balance Control in Young and Older Adults.” *Journal of Biomechanics* 49 (7): 1264–67. doi:10.1016/j.jbiomech.2016.03.011.

Barrois, R., L. Oudre, Th Moreau, Ch Truong, N. Vayatis, S. Buffat, A. Yelnik, et al. 2015. “Quantify Osteoarthritis Gait at the Doctor’s Office: A Simple Pelvis Accelerometer Based Method Independent from Footwear and Aging.” *Computer Methods in Biomechanics and Biomedical Engineering* 18 Suppl 1: 1880–81. doi:10.1080/10255842.2015.1072414.

Bolink, S. A. A. N., B. Grimm, and I. C. Heyligers. 2015. “Patient-Reported Outcome Measures versus Inertial Performance-Based Outcome Measures: A Prospective Study in Patients Undergoing Primary Total Knee Arthroplasty.” *The Knee* 22 (6): 618–23. doi:10.1016/j.knee.2015.04.002.

Bolink, S. A. A. N., E. Lenguerrand, L. R. Brunton, V. Wylde, R. Gooberman-Hill, I. C. Heyligers, A. W. Blom, and B. Grimm. 2016. “Assessment of Physical Function Following Total Hip Arthroplasty: Inertial Sensor Based Gait Analysis Is Supplementary to Patient-Reported Outcome Measures.” *Clinical Biomechanics (Bristol, Avon)* 32 (February): 171–79. doi:10.1016/j.clinbiomech.2015.11.014.

Bolink, Stijn A. A. N., Luke R. Brunton, Simon van Laarhoven, Matthijs Lipperts, Ide C. Heyligers, Ashley W. Blom, and Bernd Grimm. 2015. “Frontal Plane Pelvic Motion during Gait Captures Hip Osteoarthritis Related Disability.” *Hip International : The Journal of Clinical and Experimental Research on Hip Pathology and Therapy* 25 (5): 413–19. doi:10.5301/hipint.5000282.

Bragge, Timo, Tarja Lyytinen, Marko Hakkarainen, Paavo Vartiainen, Tuomas Liikavainio, Pasi A. Karjalainen, and Jari P. Arokoski. 2014. “Lower Impulsive Loadings Following Intensive Weight Loss after Bariatric Surgery in Level and Stair Walking: A Preliminary Study.” *The Knee* 21 (2): 534–40. doi:10.1016/j.knee.2013.11.012.

Bregou Bourgeois, A., B. Mariani, K. Aminian, P. Y. Zambelli, and C. J. Newman. 2014. “Spatio-Temporal Gait Analysis in Children with Cerebral Palsy Using, Foot-Worn Inertial Sensors.” *Gait & Posture* 39 (1): 436–42. doi:10.1016/j.gaitpost.2013.08.029.

Brodie, Matthew A. D., Colleen G. Canning, Tim R. Beijer, and Stephen R. Lord. 2015. “Uncontrolled Head Oscillations in People with Parkinson’s Disease May Reflect an Inability to Respond to Perturbations While Walking.” *Physiological Measurement* 36 (5): 873–81. doi:10.1088/0967-3334/36/5/873.

Brodie, Matthew A. D., Hylton B. Menz, Stuart T. Smith, Kim Delbaere, and Stephen R. Lord. 2015. “Good Lateral Harmonic Stability Combined with Adequate Gait Speed Is Required for Low Fall Risk in Older People.” *Gerontology* 61 (1): 69–78. doi:10.1159/000362836.

Brodie, Matthew A., Stephen R. Lord, Milou J. Coppens, Janneke Annegarn, and Kim Delbaere. 2015. “Eight-Week Remote Monitoring Using a Freely Worn Device Reveals Unstable Gait Patterns in Older Fallers.” *IEEE Transactions on Bio-Medical Engineering* 62 (11): 2588–94. doi:10.1109/TBME.2015.2433935.

Buckinx, F., C. Beaudart, J. Slomian, D. Maquet, M. Demonceau, S. Gillain, J. Petermans, J. Y. Reginster, and O. Bruyere. 2015. “Added Value of a Triaxial Accelerometer Assessing Gait Parameters to Predict Falls and Mortality among Nursing Home Residents: A Two-Year Prospective Study.” *Technology and Health Care : Official Journal of the European Society for Engineering and Medicine* 23 (2): 195–203. doi:10.3233/THC-140883.

Chini, Giorgia, Alberto Ranavolo, Francesco Draicchio, Carlo Casali, Carmela Conte, Giovanni Martino, Luca Leonardi, et al. 2016. “Local Stability of the Trunk in Patients with Degenerative Cerebellar Ataxia During Walking.” *Cerebellum (London, England)*, January. doi:10.1007/s12311-016-0760-6.

Chopra, S, H Rouhani, M Assal, K Aminian, and X Crevoisier. 2014. “Outcome of Unilateral Ankle Arthrodesis and Total Ankle Replacement in Terms of Bilateral Gait Mechanics.” *Journal of Orthopaedic Research* 32 (3): 377–84.

Collett, Johnny, Patrick Esser, Hanan Khalil, Monica Busse, Lori Quinn, Katy DeBono, Anne Rosser, Andrea H. Nemeth, and Helen Dawes. 2014. “Insights into Gait Disorders: Walking Variability Using Phase Plot Analysis, Huntington’s Disease.” *Gait & Posture* 40 (4): 694–700. doi:10.1016/j.gaitpost.2014.08.001.

Cui, Xingran, Chung-Kang Peng, Madalena D. Costa, Aner Weiss, Ary L. Goldberger, and Jeffrey M. Hausdorff. 2014. “Development of a New Approach to Quantifying Stepping Stability Using Ensemble Empirical Mode Decomposition.” *Gait & Posture* 39 (1): 495–500. doi:10.1016/j.gaitpost.2013.08.036.

Del Din, Silvia, Alan Godfrey, Brook Galna, Sue Lord, and Lynn Rochester. 2016. “Free-Living Gait Characteristics in Ageing and Parkinson’s Disease: Impact of Environment and Ambulatory Bout Length.” *Journal of Neuroengineering and Rehabilitation* 13 (1): 46. doi:10.1186/s12984-016-0154-5.

Del Din, Silvia, Alan Godfrey, and Lynn Rochester. 2015. “Validation of an Accelerometer to Quantify a Comprehensive Battery of Gait Characteristics in Healthy Older Adults and Parkinson’s Disease: Toward Clinical and at Home Use.” *IEEE Journal of Biomedical and Health Informatics*, April. doi:10.1109/JBHI.2015.2419317.

Demonceau, Marie, Anne-Francoise Donneau, Jean-Louis Croisier, Eva Skawiniak, Mohamed Boutaayamou, Didier Maquet, and Gaetan Garraux. 2015. “Contribution of a Trunk Accelerometer System to the Characterization of Gait in Patients With Mild-to-Moderate Parkinson’s Disease.” *IEEE Journal of Biomedical and Health Informatics* 19 (6): 1803–8. doi:10.1109/JBHI.2015.2469540.

Doi, Takehiko, Hiroyuki Shimada, Hyuma Makizako, Kota Tsutsumimoto, Ryo Hotta, Sho Nakakubo, and Takao Suzuki. 2015. “Effects of White Matter Lesions on Trunk Stability during Dual-Task Walking among Older Adults with Mild Cognitive Impairment.” *Age (Dordrecht, Netherlands)* 37 (6): 120. doi:10.1007/s11357-015-9858-x.

Elbaz, Avi, Amit Mor, Ganit Segal, Dana Bar, Maureen K. Monda, Benjamin Kish, Meir Nyska, and Ezequiel Palmanovich. 2016. “Lower Extremity Kinematic Profile of Gait of Patients After Ankle Fracture: A Case-Control Study.” *The Journal of Foot and Ankle Surgery : Official Publication of the American College of Foot and Ankle Surgeons*, June. doi:10.1053/j.jfas.2016.04.004.

Ellis, Robert J., Yee Sien Ng, Shenggao Zhu, Dawn M. Tan, Boyd Anderson, Gottfried Schlaug, and Ye Wang. 2015. “A Validated Smartphone-Based Assessment of Gait and Gait Variability in Parkinson’s Disease.” *PloS One* 10 (10): e0141694. doi:10.1371/journal.pone.0141694.

Galan-Mercant, Alejandro, and Antonio I. Cuesta-Vargas. 2014. “Differences in Trunk Accelerometry between Frail and Non-Frail Elderly Persons in Functional Tasks.” *BMC Research Notes* 7: 100. doi:10.1186/1756-0500-7-100.

Gillain, S., M. Drame, F. Lekeu, V. Wojtasik, C. Ricour, J.-L. Croisier, E. Salmon, and J. Petermans. 2016. “Gait Speed or Gait Variability, Which One to Use as a Marker of Risk to Develop Alzheimer Disease? A Pilot Study.” *Aging Clinical and Experimental Research* 28 (2): 249–55. doi:10.1007/s40520-015-0392-6.

Greene, Barry R., Emer P. Doheny, Aisling O’Halloran, and Rose Anne Kenny. 2014. “Frailty Status Can Be Accurately Assessed Using Inertial Sensors and the TUG Test.” *Age and Ageing* 43 (3): 406–11. doi:10.1093/ageing/aft176.

Hatanaka, Noriko, Kota Sato, Nozomi Hishikawa, Mami Takemoto, Yasuyuki Ohta, Toru Yamashita, and Koji Abe. 2016. “Comparative Gait Analysis in Progressive Supranuclear Palsy and Parkinson’s Disease.” *European Neurology* 75 (5–6): 282–89. doi:10.1159/000445111.

Henchoz, Yves, Nicola Soldini, Nicolas Peyrot, and Davide Malatesta. 2015. “Energetics and Mechanics of Walking in Patients with Chronic Low Back Pain and Healthy Matched Controls.” *European Journal of Applied Physiology* 115 (11): 2433–43. doi:10.1007/s00421-015-3227-4.

Henderson, Emily J., Stephen R. Lord, Matthew A. Brodie, Daisy M. Gaunt, Andrew D. Lawrence, Jacqueline C. T. Close, A. L. Whone, and Y. Ben-Shlomo. 2016. “Rivastigmine for Gait Stability in Patients with Parkinson’s Disease (ReSPonD): A Randomised, Double-Blind, Placebo-Controlled, Phase 2 Trial.” *The Lancet. Neurology* 15 (3): 249–58. doi:10.1016/S1474-4422(15)00389-0.

Hjorth, M. H., M. Stilling, N. D. Lorenzen, S. S. Jakobsen, K. Soballe, and I. Mechlenburg. 2014. “Block-Step Asymmetry 5 Years after Large-Head Metal-on-Metal Total Hip Arthroplasty Is Related to Lower Muscle Mass and Leg Power on the Implant Side.” *Clinical Biomechanics (Bristol, Avon)* 29 (6): 684–90. doi:10.1016/j.clinbiomech.2014.03.007.

Howcroft, Jennifer D., Edward D. Lemaire, Jonathan Kofman, and William E. McIlroy. 2014. “Analysis of Dual-Task Elderly Gait Using Wearable Plantar-Pressure Insoles and Accelerometer.” *Conference Proceedings : ... Annual International Conference of the IEEE Engineering in Medicine and Biology Society. IEEE Engineering in Medicine and Biology Society. Annual Conference* 2014: 5003–6. doi:10.1109/EMBC.2014.6944748.

Howcroft, Jennifer, Jonathan Kofman, Edward D. Lemaire, and William E. McIlroy. 2016. “Analysis of Dual-Task Elderly Gait in Fallers and Non-Fallers Using Wearable Sensors.” *Journal of Biomechanics* 49 (7): 992–1001. doi:10.1016/j.jbiomech.2016.01.015.

Howell, David, Louis Osternig, and Li-Shan Chou. 2015. “Monitoring Recovery of Gait Balance Control Following Concussion Using an Accelerometer.” *Journal of Biomechanics* 48 (12): 3364–68. doi:10.1016/j.jbiomech.2015.06.014.

Hsu, Yu-Liang, Pau-Choo Julia Chung, Wei-Hsin Wang, Ming-Chyi Pai, Chun-Yao Wang, Chien-Wen Lin, Hao-Li Wu, and Jeen-Shing Wang. 2014. “Gait and Balance Analysis for Patients with Alzheimer’s Disease Using an Inertial-Sensor-Based Wearable Instrument.” *IEEE Journal of Biomedical and Health Informatics* 18 (6): 1822–30. doi:10.1109/JBHI.2014.2325413.

Ihlen, Espen A. F., Aner Weiss, Yoav Beck, Jorunn L. Helbostad, and Jeffrey M. Hausdorff. 2016. “A Comparison Study of Local Dynamic Stability Measures of Daily Life Walking in Older Adult Community-Dwelling Fallers and Non-Fallers.” *Journal of Biomechanics* 49 (9): 1498–1503. doi:10.1016/j.jbiomech.2016.03.019.

Ihlen, Espen A. F., Aner Weiss, Alan Bourke, Jorunn L. Helbostad, and Jeffrey M. Hausdorff. 2016. “The Complexity of Daily Life Walking in Older Adult Community-Dwelling Fallers and Non-Fallers.” *Journal of Biomechanics* 49 (9): 1420–28. doi:10.1016/j.jbiomech.2016.02.055.

Jaywant, Abhishek, Terry D. Ellis, Serge Roy, Cheng-Chieh Lin, Sandy Neargarder, and Alice Cronin-Golomb. 2016. “Randomized Controlled Trial of a Home-Based Action Observation Intervention to Improve Walking in Parkinson Disease.” *Archives of Physical Medicine and Rehabilitation* 97 (5): 665–73. doi:10.1016/j.apmr.2015.12.029.

Kleiner, Ana, Manuela Galli, Maria Gaglione, Daniela Hildebrand, Patrizio Sale, Giorgio Albertini, Fabrizio Stocchi, and Maria Francesca De Pandis. 2015. “The Parkinsonian Gait Spatiotemporal Parameters Quantified by a Single Inertial Sensor before and after Automated Mechanical Peripheral Stimulation Treatment.” *Parkinson’s Disease* 2015: 390512. doi:10.1155/2015/390512.

Kobsar, Dylan, Chad Olson, Raman Paranjape, Thomas Hadjistavropoulos, and John M. Barden. 2014. “Evaluation of Age-Related Differences in the Stride-to-Stride Fluctuations, Regularity and Symmetry of Gait Using a Waist-Mounted Tri-Axial Accelerometer.” *Gait & Posture* 39 (1): 553–57. doi:10.1016/j.gaitpost.2013.09.008.

Lauretani, Fulvio, Laura Galuppo, Cosimo Costantino, Andrea Ticinesi, Gianpaolo Ceda, Livio Ruffini, Anna Nardelli, and Marcello Maggio. 2016. “Parkinson’s Disease (PD) with Dementia and Falls Is Improved by AChEI? A Preliminary Study Report.” *Aging Clinical and Experimental Research* 28 (3): 551–55. doi:10.1007/s40520-015-0437-x.

Mancini, Martina, Lorenzo Chiari, Lars Holmstrom, Arash Salarian, and Fay B. Horak. 2016. “Validity and Reliability of an IMU-Based Method to Detect APAs prior to Gait Initiation.” *Gait & Posture* 43 (January): 125–31. doi:10.1016/j.gaitpost.2015.08.015.

Martinez-Ramirez, Alicia, Ion Martinikorena, Marisol Gomez, Pablo Lecumberri, Nora Millor, Leocadio Rodriguez-Manas, Francisco Jose Garcia Garcia, and Mikel Izquierdo. 2015. “Frailty Assessment Based on Trunk Kinematic Parameters during Walking.” *Journal of Neuroengineering and Rehabilitation* 12: 48. doi:10.1186/s12984-015-0040-6.

Martinez-Ramirez, Alicia, Ion Martinikorena, Pablo Lecumberri, Marisol Gomez, Nora Millor, Alvaro Casas-Herrero, Fabricio Zambom-Ferraresi, and Mikel Izquierdo. 2016. “Dual Task Gait Performance in Frail Individuals with and without Mild Cognitive Impairment.” *Dementia and Geriatric Cognitive Disorders* 42 (1–2): 7–16. doi:10.1159/000447451.

Martinikorena, Ion, Alicia Martinez-Ramirez, Marisol Gomez, Pablo Lecumberri, Alvaro Casas-Herrero, Eduardo L. Cadore, Nora Millor, Fabricio Zambom-Ferraresi, Fernando Idoate, and Mikel Izquierdo. 2016. “Gait Variability Related to Muscle Quality and Muscle Power Output in Frail Nonagenarian Older Adults.” *Journal of the American Medical Directors Association* 17 (2): 162–67. doi:10.1016/j.jamda.2015.09.015.

Martino Cinnera, Alex, S. Bonni, M. Iosa, V. Ponzo, A. Fusco, Carlo Caltagirone, and Giacomo Koch. 2015. “Clinical Effects of Non-Invasive Cerebellar Magnetic Stimulation Treatment Combined with Neuromotor Rehabilitation in Traumatic Brain Injury. A Single Case Study.” *Functional Neurology*, May, 1–4.

Matsumoto, Hiromi, Hiroshi Hagino, Mari Osaki, Shinji Tanishima, Chika Tanimura, Akihiro Matsuura, and Tomoyuki Makabe. 2016. “Gait Variability Analysed Using an Accelerometer Is Associated with Locomotive Syndrome among the General Elderly Population: The GAINA Study.” *Journal of Orthopaedic Science : Official Journal of the Japanese Orthopaedic Association* 21 (3): 354–60. doi:10.1016/j.jos.2016.02.003.

Matsushima, Akira, Kunihiro Yoshida, Hirokazu Genno, Asuka Murata, Setsuko Matsuzawa, Katsuya Nakamura, Akinori Nakamura, and Shu-Ichi Ikeda. 2015. “Clinical Assessment of Standing and Gait in Ataxic Patients Using a Triaxial Accelerometer.” *Cerebellum & Ataxias* 2: 9. doi:10.1186/s40673-015-0028-9.

Mirelman, Anat, Aner Weiss, Aron S. Buchman, David A. Bennett, Nir Giladi, and Jefferey M. Hausdorff. 2014. “Association between Performance on Timed Up and Go Subtasks and Mild Cognitive Impairment: Further Insights into the Links between Cognitive and Motor Function.” *Journal of the American Geriatrics Society* 62 (4): 673–78. doi:10.1111/jgs.12734.

Motta, Caterina, Eduardo Palermo, Valeria Studer, Marco Germanotta, Giorgio Germani, Diego Centonze, Paolo Cappa, Silvia Rossi, and Stefano Rossi. 2016. “Disability and Fatigue Can Be Objectively Measured in Multiple Sclerosis.” *PloS One* 11 (2): e0148997. doi:10.1371/journal.pone.0148997.

Pan, Di, Rohit Dhall, Abraham Lieberman, and Diana B. Petitti. 2015. “A Mobile Cloud-Based Parkinson’s Disease Assessment System for Home-Based Monitoring.” *JMIR mHealth and uHealth* 3 (1): e29. doi:10.2196/mhealth.3956.

Patterson, Matthew R., Eamonn Delahunt, Kevin T. Sweeney, and Brian Caulfield. 2014. “An Ambulatory Method of Identifying Anterior Cruciate Ligament Reconstructed Gait Patterns.” *Sensors (Basel, Switzerland)* 14 (1): 887–99. doi:10.3390/s140100887.

Pau, Massimiliano, Bruno Leban, Giorgia Collu, and Gian Mario Migliaccio. 2014. “Effect of Light and Vigorous Physical Activity on Balance and Gait of Older Adults.” *Archives of Gerontology and Geriatrics* 59 (3): 568–73. doi:10.1016/j.archger.2014.07.008.

Perrochon, Anaick, Achille E. Tchalla, Joelle Bonis, Florian Perucaud, and Stephane Mandigout. 2015. “Effects of a Multicomponent Exercise Program on Spatiotemporal Gait Parameters, Risk of Falling and Physical Activity in Dementia Patients.” *Dementia and Geriatric Cognitive Disorders Extra* 5 (3): 350–60. doi:10.1159/000435772.

Rahman, J., Q. Tang, M. Monda, J. Miles, and I. McCarthy. 2015. “Gait Assessment as a Functional Outcome Measure in Total Knee Arthroplasty: A Cross-Sectional Study.” *BMC Musculoskeletal Disorders* 16 (1). http://www.embase.com/search/results?subaction=viewrecord&from=export&id=L603257810.

Rapp, Walter, Torsten Brauner, Linda Weber, Stefan Grau, Annegret Mundermann, and Thomas Horstmann. 2015. “Improvement of Walking Speed and Gait Symmetry in Older Patients after Hip Arthroplasty: A Prospective Cohort Study.” *BMC Musculoskeletal Disorders* 16: 291. doi:10.1186/s12891-015-0755-3.

Reginatto, Brenda, Kenneth Taylor, Matthew R. Patterson, Dermot Power, Yusuke Komaba, Kazuho Maeda, Akihiro Inomata, and Brian Caulfield. 2015. “Context Aware Falls Risk Assessment: A Case Study Comparison.” *Conference Proceedings : ... Annual International Conference of the IEEE Engineering in Medicine and Biology Society. IEEE Engineering in Medicine and Biology Society. Annual Conference* 2015: 5477–80. doi:10.1109/EMBC.2015.7319631.

Reynard, Fabienne, Philippe Vuadens, Olivier Deriaz, and Philippe Terrier. 2014. “Could Local Dynamic Stability Serve as an Early Predictor of Falls in Patients with Moderate Neurological Gait Disorders? A Reliability and Comparison Study in Healthy Individuals and in Patients with Paresis of the Lower Extremities.” *PloS One* 9 (6): e100550. doi:10.1371/journal.pone.0100550.

Rivolta, Massimo W., Md Aktaruzzaman, Giovanna Rizzo, Claudio L. Lafortuna, Maurizio Ferrarin, Gabriele Bovi, Daniela R. Bonardi, and Roberto Sassi. 2015. “Automatic vs. Clinical Assessment of Fall Risk in Older Individuals: A Proof of Concept.” *Conference Proceedings : ... Annual International Conference of the IEEE Engineering in Medicine and Biology Society. IEEE Engineering in Medicine and Biology Society. Annual Conference* 2015 (August): 6935–38. doi:10.1109/EMBC.2015.7319987.

Saether, Rannei, Jorunn L. Helbostad, Lars Adde, Siri Braendvik, Stian Lydersen, and Torstein Vik. 2014. “Gait Characteristics in Children and Adolescents with Cerebral Palsy Assessed with a Trunk-Worn Accelerometer.” *Research in Developmental Disabilities* 35 (7): 1773–81. doi:10.1016/j.ridd.2014.02.011.

———. 2015. “The Relationship between Trunk Control in Sitting and during Gait in Children and Adolescents with Cerebral Palsy.” *Developmental Medicine and Child Neurology* 57 (4): 344–50. doi:10.1111/dmcn.12628.

Scaglioni-Solano, P, and LF Aragon-Vargas. 2015. “Age-Related Differences When Walking Downhill on Different Sloped Terrains.” *Gait & Posture* 41 (1): 153–58.

Schmitz-Hubsch, Tanja, Alexander U. Brandt, Caspar Pfueller, Leonora Zange, Adrian Seidel, Andrea A. Kuhn, Friedemann Paul, Martina Minnerop, and Sarah Doss. 2016. “Accuracy and Repeatability of Two Methods of Gait Analysis - GaitRite Und Mobility Lab - in Subjects with Cerebellar Ataxia.” *Gait & Posture* 48 (May): 194–201. doi:10.1016/j.gaitpost.2016.05.014.

Schooten, Kimberley S. van, Mirjam Pijnappels, Sietse M. Rispens, Petra J. M. Elders, Paul Lips, Andreas Daffertshofer, Peter J. Beek, and Jaap H. van Dieën. 2016. “Daily-Life Gait Quality as Predictor of Falls in Older People: A 1-Year Prospective Cohort Study.” *PloS One* 11 (7): e0158623. doi:10.1371/journal.pone.0158623.

Sejdic, Ervin, Kristin A. Lowry, Jennica Bellanca, Mark S. Redfern, and Jennifer S. Brach. 2014. “A Comprehensive Assessment of Gait Accelerometry Signals in Time, Frequency and Time-Frequency Domains.” *IEEE Transactions on Neural Systems and Rehabilitation Engineering : A Publication of the IEEE Engineering in Medicine and Biology Society* 22 (3): 603–12. doi:10.1109/TNSRE.2013.2265887.

Sheehan, K. J., B. R. Greene, C. Cunningham, L. Crosby, and R. A. Kenny. 2014. “Early Identification of Declining Balance in Higher Functioning Older Adults, an Inertial Sensor Based Method.” *Gait & Posture* 39 (4): 1034–39. doi:10.1016/j.gaitpost.2014.01.003.

Shin, Sun-Shil, Duk-Hyun An, and Won-Gyu Yoo. 2015. “Comparison of Gait Velocity and Center of Mass during Square and Semicircular Turning Gaits between Groups of Elderly People with Differing Visual Acuity.” *Journal of Physical Therapy Science* 27 (2): 387–88. doi:10.1589/jpts.27.387.

Shirai, Shinichi, Ichiro Yabe, Masaaki Matsushima, Yoichi M. Ito, Mitsuru Yoneyama, and Hidenao Sasaki. 2015. “Quantitative Evaluation of Gait Ataxia by Accelerometers.” *Journal of the Neurological Sciences* 358 (1–2): 253–58. doi:10.1016/j.jns.2015.09.004.

Staab, Wieland, Ralf Hottowitz, Christian Sohns, Jan Martin Sohns, Fabian Gilbert, Jan Menke, Andree Niklas, and Joachim Lotz. 2014. “Accelerometer and Gyroscope Based Gait Analysis Using Spectral Analysis of Patients with Osteoarthritis of the Knee.” *Journal of Physical Therapy Science* 26 (7): 997–1002. doi:10.1589/jpts.26.997.

Summa, Aurora, Giuseppe Vannozzi, Elena Bergamini, Marco Iosa, Daniela Morelli, and Aurelio Cappozzo. 2016. “Multilevel Upper Body Movement Control during Gait in Children with Cerebral Palsy.” *PloS One* 11 (3): e0151792. doi:10.1371/journal.pone.0151792.

Tadano, Shigeru, Ryo Takeda, Keita Sasaki, Tadashi Fujisawa, and Harukazu Tohyama. 2016. “Gait Characterization for Osteoarthritis Patients Using Wearable Gait Sensors (H-Gait Systems).” *Journal of Biomechanics* 49 (5): 684–90. doi:10.1016/j.jbiomech.2016.01.017.

Toebes, Marcel J. P., Marco J. M. Hoozemans, Regula Furrer, Joost Dekker, and Jaap H. van Dieen. 2015. “Associations between Measures of Gait Stability, Leg Strength and Fear of Falling.” *Gait & Posture* 41 (1): 76–80. doi:10.1016/j.gaitpost.2014.08.015.

Valenti, Giulio, Alberto G. Bonomi, and Klaas R. Westerterp. 2015. “Body Acceleration as Indicator for Walking Economy in an Ageing Population.” *PloS One* 10 (10): e0141431. doi:10.1371/journal.pone.0141431.

Weiss, Aner, Talia Herman, Nir Giladi, and Jeffrey M. Hausdorff. 2014. “Objective Assessment of Fall Risk in Parkinson’s Disease Using a Body-Fixed Sensor Worn for 3 Days.” *PloS One* 9 (5): e96675. doi:10.1371/journal.pone.0096675.

———. 2015a. “Association between Community Ambulation Walking Patterns and Cognitive Function in Patients with Parkinson’s Disease: Further Insights into Motor-Cognitive Links.” *Parkinson’s Disease* 2015: 547065. doi:10.1155/2015/547065.

———. 2015b. “New Evidence for Gait Abnormalities among Parkinson’s Disease Patients Who Suffer from Freezing of Gait: Insights Using a Body-Fixed Sensor Worn for 3 Days.” *Journal of Neural Transmission (Vienna, Austria : 1996)* 122 (3): 403–10. doi:10.1007/s00702-014-1279-y.

Yoneyama, Mitsuru, Hiroshi Mitoma, Maya Higuma, Nobuo Sanjo, Takanori Yokota, and Hiroo Terashi. 2015. “Ambulatory Gait Behavior in Patients with Dementia: A Comparison with Parkinson’s Disease.” *IEEE Transactions on Neural Systems and Rehabilitation Engineering : A Publication of the IEEE Engineering in Medicine and Biology Society*, September. doi:10.1109/TNSRE.2015.2477856.

Zakaria, Nor Aini, Yutaka Kuwae, Toshiyo Tamura, Kotaro Minato, and Shigehiko Kanaya. 2015. “Quantitative Analysis of Fall Risk Using TUG Test.” *Computer Methods in Biomechanics and Biomedical Engineering* 18 (4): 426–37. doi:10.1080/10255842.2013.805211.

Zollinger, Marie, Francis Degache, Gabriel Currat, Ludmila Pochon, Nicolas Peyrot, Christopher J. Newman, and Davide Malatesta. 2016. “External Mechanical Work and Pendular Energy Transduction of Overground and Treadmill Walking in Adolescents with Unilateral Cerebral Palsy.” *Frontiers in Physiology* 7: 121. doi:10.3389/fphys.2016.00121.
